# Immunomodulation by the Commensal Microbiome During Immune-Targeted Interventions: Focus on Cancer Immune Checkpoint Inhibitor Therapy and Vaccination

**DOI:** 10.3389/fimmu.2021.643255

**Published:** 2021-05-13

**Authors:** Abigail L. Reens, Damien J. Cabral, Xue Liang, James E. Norton, Alex G. Therien, Daria J. Hazuda, Gokul Swaminathan

**Affiliations:** ^1^ Exploratory Science Center, Merck & Co., Inc., Cambridge, MA, United States; ^2^ Infectious Disease and Vaccine Research, Merck & Co., Inc., West Point, PA, United States

**Keywords:** microbiome, immune checkpoint inhibitors, vaccines, innate immunity, immuno-oncology, adaptive immunity

## Abstract

Emerging evidence in clinical and preclinical studies indicates that success of immunotherapies can be impacted by the state of the microbiome. Understanding the role of the microbiome during immune-targeted interventions could help us understand heterogeneity of treatment success, predict outcomes, and develop additional strategies to improve efficacy. In this review, we discuss key studies that reveal reciprocal interactions between the microbiome, the immune system, and the outcome of immune interventions. We focus on cancer immune checkpoint inhibitor treatment and vaccination as two crucial therapeutic areas with strong potential for immunomodulation by the microbiota. By juxtaposing studies across both therapeutic areas, we highlight three factors prominently involved in microbial immunomodulation: short-chain fatty acids, microbe-associate molecular patterns (MAMPs), and inflammatory cytokines. Continued interrogation of these models and pathways may reveal critical mechanistic synergies between the microbiome and the immune system, resulting in novel approaches designed to influence the efficacy of immune-targeted interventions.

## Introduction: The Microbiome and the Immune System

**Figure 1:**
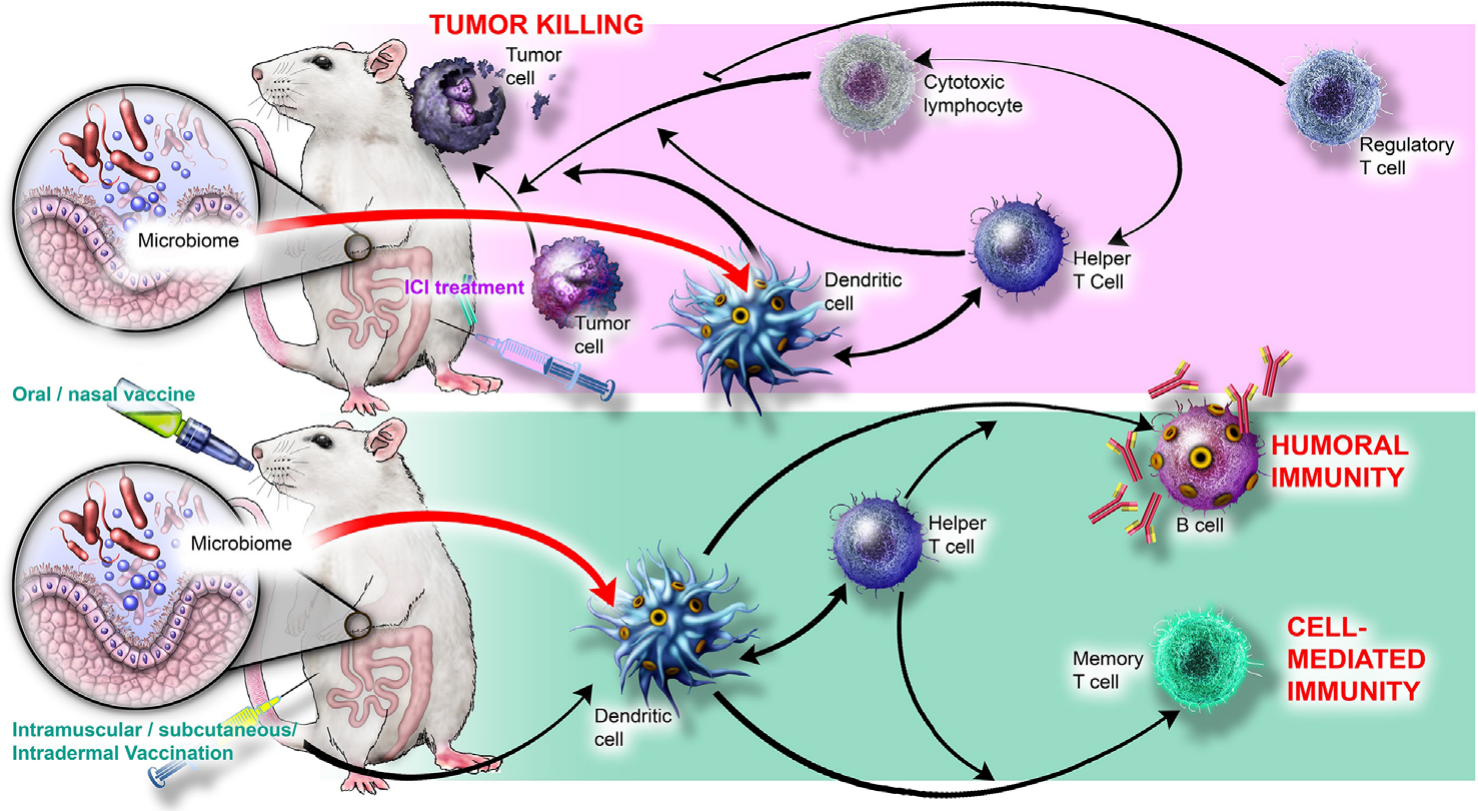
Cellular players and interactions involved in immune checkpoint inhibitor-mediated tumor killing and vaccine-induced immunity. (Top) ICIs stimulate cytotoxic lymphocytes, Th1 helper T cells, and DCs to kill tumor cells; Tregs inhibit killing. MAMPs produced by bacteria such as *B. thetaiotaomicron* and *B. fragilis* may interact with TLR2 and TLR4 on DCs and stimulate Th1 polarization and synergize with ICI activity. Microbial metabolites such as inosine (produced by *B. pseudolongum)* may also contribute to ICI efficacy by stimulating T cell proliferation. (Bottom) Live, inactivated, or molecular vaccination ultimately activates DCs and helper T cells to yield humoral immunity (B cell antibodies) and cell-mediated immunity (memory T cells). MAMPs, such as flagellin and peptidoglycan, interact with PRRs to stimulate B cells and Tfh cells, thereby augmenting vaccine response. Microbially produced SCFAs may also stimulate DCs.

Humans are colonized by trillions of microbes collectively termed the microbiome, consisting of bacteria, archaea, viruses, fungi, and protists (1). Together, these interrelated microbial communities represent a rich source of metabolites and ligands that broadly influence human biology, including nutrient digestion, tissue homeostasis, neuroendocrine signaling, and the development and maintenance of the immune system (reviewed in 2–4). It is becoming clear that the microbiome modulates diverse immune processes, from defense against infection (5–7) to antibody production (8, 9), and from inflammation (10–12) to autoimmunity and allergy (13–15). Immune-microbe interactions also directly regulate homeostasis and development of immune cells such as antigen-presenting cells (16–19) and T cells (20–24). Importantly, the microbiome significantly influences the host response to immune-targeted interventions (25–31), which harness the immune system to treat or prevent diseases including infections, allergy, autoimmunity, inflammatory disorders, and cancer. Two of the most prominent immune interventions currently employed in the clinic are cancer immune checkpoint inhibitors and vaccines, which together are the focus of this review; key cellular players and interactions involved in immune checkpoint inhibitor-mediated tumor killing and vaccine-induced immunity are presented in **Figure 1**.

In the past decade, immune checkpoint inhibitors (ICIs) have become an essential pillar of treatment for numerous cancers (32–34). Currently employed ICIs are monoclonal antibodies that block specific immune checkpoint receptors (CTLA-4, PD-1) or ligands (PD-L1) on the surface of lymphocytes or tumor cells, respectively (32, 34). Normally, immune checkpoint signaling prevents development of hyperactive immune responses and thus damage to healthy tissues (32). However, these checkpoints are exploited by tumor cells to evade immunosurveillance, block anti-tumor cytotoxic T lymphocytes (CTLs), and induce immunosuppressive regulatory T cells (Tregs) (35, 36). By blocking immune checkpoint receptor-ligand interactions, ICIs can restore endogenous anti-tumor immune responses and disrupt cancer progression. However, not all individuals respond to ICIs, and recent evidence suggests the microbiota may play a role in ICI responsiveness by modulating the immune system, particularly the abundances and functions of NK cells, CTLs, and Tregs (37–43).

Equally consequential, vaccines have revolutionized our ability to prevent a myriad of infectious diseases and have had a long-standing impact on global human health (44, 45). During vaccination, individuals are exposed to a foreign antigen, sometimes in the presence of an exogenous adjuvant, to activate the immune system. Immunization leads to development of immune memory: molecules and cells that are able to recognize and eliminate the corresponding pathogen before infection can be established (46). Protection is often mediated by humoral production of antigen-specific antibodies produced by B cells, though cell-mediated protection by T cells is also important for protection elicited by certain vaccines (47). Orchestration of vaccine-specific humoral or cell-mediated immunity requires finely-tuned interactions between antigen-presenting cells (APCs), B cells, and T cells (48–51). However, these cellular interactions may be predisposed to particular responses, which subsequently influence vaccine outcome, depending on the underlying immune state. This “immune tone” comprises the cytokine milieu, basal expression of surface proteins that mediate cell-cell interactions, and antigen presentation by APCs, all of which can be influenced by the microbiome (4, 52–54).

In this review, we summarize key findings in the literature that demonstrate the effect of the microbiome on outcomes of immune interventions, with a focus on ICI treatment and vaccination as the most studied examples. We discuss evidence that immunotherapies can influence the microbiota and that age plays a role in the effects of the microbiota on the immune system, as well as discuss the effects of live biotherapeutic products and prebiotics. We highlight studies that identify cellular and molecular mechanisms by which the microbiome modulates the immune system during immune interventions. Taken together, these studies reveal common microbial and immune elements across both ICI treatment and vaccination that have the potential to shape immune responses across diverse therapeutic spheres.

## Linking the Microbiome and Response to Immune Checkpoint Inhibitor Treatment

Immune checkpoint inhibitor (ICI) therapy can improve long-term outcomes in a number of different cancer types, such as melanoma, non-small cell lung cancer (NSCLC), and urothelial cancer. However, the majority of patients experience cancer recurrence or do not respond to treatment (55). A number of factors that are partially predictive of ICI responsiveness have been identified (reviewed in 56, 57). Recently, the gut microbiome, which is known to play a role in the development and function of the immune system, has also been suggested as a determinant of ICI efficacy (58–63). As a result, these studies have sparked interest in the gut microbiome as both a diagnostic and therapeutic target in the context of cancer immunotherapy. In the following sections, we review the body of clinical and preclinical studies that demonstrate a role for the gut microbiome in ICI responsiveness. We highlight potential microbial, molecular, and immune mechanisms by which the microbiome may influence response to ICI treatment, and discuss whether ICI treatment reciprocally modulates the microbiome.

### Clinical Findings

Given the role of the gut microbiota in the development and function of the immune system, it is unsurprising that the microbiome may influence ICI outcome measurements in the clinic. Several studies demonstrate that improved patient responses to ICI are associated with a “healthy” gut microbiome, as measured by higher diversity (61, 64). Similarly, antibiotic treatment either shortly before or during ICI therapy has been shown to influence outcomes (65–69). Conversely, other studies have found little to no impact of antibiotics on treatment outcome (70). It is likely that these discrepancies are due in large part to the wide range of antibiotic classes, cancer types, and treatment regimens captured by these clinical studies. Thus, additional work is required to validate these observations and better understand the impact of antibiotics and microbiome diversity on ICI efficacy.

A number of recent studies have identified unique microbial signatures that are associated with ICI treatment outcomes (**Table 1**). In particular, metastatic melanoma patients that respond to ICI therapy tend to have high abundances of *Faecalibacterium prausnitzii* (59–61, 64), which comprises a large proportion of the human gut microbiota and is known to influence immune function *via* production of short-chain fatty acids (SCFAs) (72–74). In addition to *F. prausnitzii*, other bacteria have been associated with ICI responses in melanoma patients, including *Gemmiger formicilis* (63), *Dorea formicigenerans* (60), and *Ruminococcus bromii* (61), all within the order Clostridia, as well as *Bacteroides thetaiotaomicron* (60), *Holdemania filiformis* (60), and the Actinobacteria *Bifidobacterium longum* and *Collinsella aerofaciens* (62), which have been shown to promote CD4+ T helper cell 1 (Th1) polarization (75). In contrast to these studies in melanoma patients, a study in a cohort of patients with non-small cell lung carcinoma, renal cell carcinoma, and urothelial carcinoma found that ICI treatment outcome was influenced by abundance of *Akkermansia muciniphilia* and *Enterococcus hirae*, suggesting that beneficial microbial signatures could be cancer specific (58). It was recently demonstrated that *E. hirae* harbors a bacteriophage encoding a MHC class I-binding protein that induces a CD8+ T cell response and cross-reacts with cancer antigens (76), which could explain the influence of *E*. *hirae* on ICI treatment. The presence or absence of key microbial taxa may enable the stratification of patient populations and predict potential ICI treatment outcomes based on microbiome composition. Additionally, these findings suggest that the identification of key bacterial species may facilitate development of adjunct therapies to improve ICI outcomes, such as fecal matter transplants (FMT) or probiotics (77–81). Together, these approaches may accelerate development of treatment regimens to improve ICI therapeutic outcomes.

### Pre-Clinical Findings

The clinical observation that the microbiome can influence ICI treatment response is generally recapitulated in pre-clinical studies of microbiome disruption or supplementation with fecal material, individual microbes, or microbial metabolites (**Table 2**). Mice with different microbial communities, obtained from different vendors, have been shown to respond differently to ICI treatment (82). Microbiota depletion *via* administration of a cocktail of broad-spectrum of antibiotics containing ampicillin, streptomycin, and colistin reduces the efficacy of anti-PD-1 and anti-CTLA-4 mAbs, both alone and in combination (58, 83, 85). Furthermore, this effect was observed in multiple tumor types and corresponding models including melanoma (RET), renal cell carcinoma (RENCA), colon cancer (CT26, MC38), and fibrosarcoma (MCA-205), suggesting that the microbiota may contribute to treatment responsiveness in a broad array of cancers (58, 83, 85).

Fecal microbiome transplant (FMT) from patients into mice has revealed differential effects of microbiota from ICI responders and non-responders, demonstrating microbiome-mediated immunomodulation and impact on ICI outcome. For example, germ-free (GF) or antibiotic-treated mice receiving responder FMT accumulated Th1-polarized cells in the tumor microenvironment following ICI treatment (58). Another study found that GF mice receiving responder FMT had higher frequencies of CTLs, while those receiving non-responder FMT had higher frequencies of immunosuppressive Tregs and Th17 cells (61). Similarly, a third study found that that mice receiving responder FMT displayed elevated levels of tumor-specific CTLs, but not Tregs (62). Together, these studies suggest that the microbiome from patient ICI-responders elicits anti-tumor immunity upon FMT, which in turn promote ICI response. FMT, as a critical research tool, not only demonstrated the direct modulatory effect of gut microbiome, but also opens the door to identify key microbes and/or their metabolites that stimulates the immune response.

Individual microbes have also been identified that promote ICI response by inducing Th1 polarization. Oral supplementation with *Bacteroides fragilis*, *B. thetaiotaomicron*, or *Burkholderia cepacia* restored ICI efficacy in antibiotic treated mice (83). In particular, *B. fragilis* potentiated ICI treatment by stimulating a Th1 response within the tumor-draining lymph nodes (83). This effect was mediated by stimulation of TLR2 and TLR4, which recognize microbe-associated molecular patterns (MAMPs), leading to the maturation of intratumoral dendritic cells (DCs). In a separate study, oral delivery of *A. muciniphila* and *E. hirae* to antibiotic-treated mice increased the incidence of central memory CD4^+^ T cells within the tumor bed, mesenteric lymph nodes, and draining lymph nodes and also induced production of IL-12 by DCs, a Th1 cytokine that plays a role in ICI response (58). Another study using mice with low baseline ICI response due to a unique microbial community found that administration of *Bifidobacterium* restored ICI response by stimulating antigen presentation by DCs and CTL activation (82). Additionally, a recent study found that *Bifidobacterium pseudolongum* was enriched in colon tumors of mice that responded to ICI treatment, and that colonization of germ-free mice with *B. pseudolongum* promoted ICI response (86). The authors found that this effect was mediated by stimulation of the adenosine A_2A_ receptor on T cells *via* microbially-produced inosine. Oral delivery of inosine promoted clearance of colon, bladder, and melanoma tumor models in combination with anti-CTLA-4 and CpG treatment.

In addition to inosine, other microbial metabolites have also been shown to mediate the effect of microbes on ICI outcomes. In one study, delivery of a consortium of 11 bacterial species isolated from healthy human fecal material improved ICI response and induced a robust expansion of IFN-γ producing CTLs (84). However, administration of the heat-killed consortium failed to recapitulate the effects, suggesting that active colonization is required. The authors found that mice receiving the live consortium displayed increased levels of mevalonate and dimethylglycine in both cecal contents and sera, which may increase CTL activation or expansion (87, 88). Interestingly, the effect of the consortium was independent of a number of key innate signaling pathways, but loss of CD103^+^ DCs or MHC class Ia was sufficient to abrogate expansion of CTLs, suggesting that the consortium may interact with tissue-resident DCs in an MHC class Ia-dependent manner to promote ICI response (84).

Elevated levels of microbiota-derived short-chain fatty acids, which are known to induce anti-inflammatory Tregs (40), have also been associated with reduced survival in ICI treatment (63). DCs isolated from butyrate-supplemented mice receiving ICI therapy displayed reduced surface expression of costimulatory molecules on APCs, suggesting that SCFAs may interfere with ICI therapy by inhibiting DC maturation within the tumor-draining lymph nodes (63). Another study found that antibiotics reduced the efficacy of ICI therapy in mice and decreased plasma levels of microbiota-derived metabolites known to be involved in glycerophospholipid metabolism and glycosylphosphatidylinositol (GPI)-anchor biosynthesis (85). The authors also observed reduced levels of the inflammatory cytokines IL-2 and IFN-γ within the tumor microenvironment, leading to the hypothesis that impaired glycerophospholipid metabolism by the microbiota could dampen anticancer immune responses by muting production of proinflammatory cytokines.

Thus far, multiple studies have implicated the microbiome in modulating response to ICI treatment. Clinically, several studies have suggested that the overall diversity of the microbiome is a key determinant of ICI responsiveness (61, 89). However, other studies have identified unique microbial signatures of responders and non-responders, suggesting that the presence or absence of key taxa may be more predictive of response (58–61, 64, 66, 83). Several bacteria identified in ICI responders have been shown to promote ICI efficacy in preclinical studies, indicating that supplementation with these strains could improve outcomes for patients undergoing ICI treatment. Recent molecular and cellular investigations have implicated several pathways by which the microbiota influence ICI treatment outcome, including SCFAs (63), inflammatory cytokines (58, 85), antigen presentation cell function (63, 82–84), and T cell polarization (58, 61, 83).

In addition to the established role of the gut microbiome in regulating immune function, there is also a growing body of evidence that bacteria present within the tumor microenvironment (collectively termed the tumor microbiome) may also impact anti-cancer responses. For example, *F. nucleatum* is known to form biofilm-like structures within tumor spheroids *in vitro* and has been shown to directly inhibit NK cells *via* engaging TIGIT (90–92). Furthermore, a recent analysis of human tumors found that different tumor types displayed unique microbial signatures and that responders to ICI had tumors containing elevated abundances of Clostridia (93). However, it is unclear what role the tumor microbiome plays as a determinant of ICI efficacy. Further investigations of the roles of these microbial communities and their impact on immune function may facilitate target-specific therapeutic approaches to promote ICI response in patients.

### Influence of Immune Checkpoint Inhibitors on the Microbiome

Immunomodulatory agents have been shown to change microbial composition, likely by modulating immune-mediated control of the microbiota (27, 94–98). Thus, an open question is whether cancer ICIs influence the microbial community. Emerging evidence suggests that ICIs targeting different immune checkpoint proteins may differentially impact the microbiome depending on their propensity to cause gastrointestinal adverse events such as diarrhea or colitis. However, the immune-microbe interactions that mediate these distinct effects are largely unknown.

Anti-PD-1/PD-L1 drugs appear to have minimal impact on the gut microbiome, in line with a lower incidence of gastrointestinal adverse events compared to other immunotherapy drugs (99). A recent study examining the gut microbiota of melanoma patients receiving anti-PD-1 therapy found no significant differences in microbial diversity or composition (61). Similarly, anti-PD-1 therapy for either renal cell carcinoma or non-small cell lung cancer had no impact on microbiome diversity or gene content after one month treatment, despite of elevation after two months (58). In contrast, anti-CTLA-4 treatment, which is known to be associated with elevated incidence of gastrointestinal adverse events, has been found to increase the abundance of *Bacteroides* genus in mice and a cohort of metastatic melanoma patients (83). Another study also found that anti-CTLA-4 treatment was associated with a reduction of multiple Firmicute species and an enrichment of *Bacteroides*, but only in patients were experiencing acute immune-related colitis (59). Lastly, combinatorial therapy with anti-PD-1 and anti-CTLA-4 has been shown to induce limited changes in the gut microbiome (60). However, the changes observed (an increase in *Bacteroides stercoris* and a reduction in *Clostridium boltae*) are largely consistent with those seen in other studies examining anti-CTLA-4 alone (59, 60, 83).

Based on these findings, it appears that major changes to the microbial community after ICI treatment are likely the secondary result of immune-related colitis, and it is unlikely that ICIs have a direct impact on microbiome composition. As a result, regimens targeting CTLA-4 are more likely to induce microbiome shifts than those targeting PD-1 due to their greater incidence of gastrointestinal adverse events. However, there are a number of limitations to the existing studies that make it difficult to draw definitive conclusions. Several of the studies have very small sample sizes (n <10), making it challenging to detect potentially small changes in microbial composition. Additionally, these studies differ considerably in methodology of microbiome sequencing and analysis, which limits our capacity to compare microbial signatures across cohorts. Further complicating comparisons between studies are considerable differences between treatment regimens, ICI dosages, and cancer types. Therefore, these limitations highlight a need for additional well-controlled and thorough studies to understand the impact of ICIs on the gut microbiome and whether ICI-induced microbiome changes subsequently influence ICI treatment outcomes.

### Microbial Manipulation During ICI Treatment

Clinical and preclinical efforts suggest a link between the state of the microbiome and ICI treatment outcomes. As described above, preclinical studies in mice have found that fecal microbiota transplant, delivery of single or consortia of microbes, or supplementation with microbial molecules can modulate ICI treatment outcomes. Whether microbiome manipulation improves ICI treatment outcomes in clinical patients is a crucial question for ongoing and future studies. In one study, fecal matter transplants (FMT) from donors that have achieved a complete response with anti-PD-1 therapy were shown to improve responsiveness to ICI in some metastatic melanoma patients (100). Additionally, FMT recipients were found to have increased expression of genes involved in the presentation of peptides on MHC-I molecules in APCs along with elevated IL-1-mediated signaling, suggesting a mechanistic link between FMT and improved ICI responsiveness in a clinical setting (100). Another recent study found that supplementation with the probiotic *Clostridium butyricum* prior to or during ICI therapy improved patient outcomes (71). Although *C. butyricum* was selectively given to patients based on symptoms of gastrointestinal upset, the effect was observed in patients with or without prior antibiotic treatment. This result suggests that promoting a normobiotic microbiome could not only relieve gastrointestinal side effects of ICI treatment as has been described (101), but may also feedback to enhance ICI treatment outcomes (102).

## Linking the Microbiome and Response to Vaccines

Vaccination confers protection against pathogens. However, the response to vaccination varies widely across individuals, which could greatly compromise individual and community protection (reviewed in 103, 104). Several factors that contribute to vaccine non-responsiveness in humans have been identified, including genetics (105, 106), advanced age (reviewed in 107–109), smoking (110), and comorbidities such as infection (111–113), obesity (114), malnutrition (115), kidney disease (116), and autoimmune disorders or allergy (117, 118).

Recently, clinical and preclinical studies have suggested microbial modulation of the immune system is directly responsible for the effects of the microbiome on vaccine response (summarized in **Table 3**). It is also possible the microbiome is the mediator of other factors associated with vaccine nonresponse, for example obesity or celiac disease, which are known to induce changes in the microbiome (147, 148). Thus, manipulation of the microbiota or direct targeting of microbially-regulated immune pathways could represent attractive strategies for promoting vaccine response in the broad healthy population or specific sub-populations with characteristically poor response (4, 147, 149–153).

In the following sections, we review key clinical and preclinical studies that link the microbiota and vaccine outcome, and we highlight putative immunomodulatory mechanisms by which the microbiome may influence vaccine responsiveness. We also discuss how the connections between the microbiome and vaccine response evolve over the course of a lifetime, and describe current approaches to harness the microbiota to promote vaccine response.

### Clinical Findings

Within the last two decades, observational clinical studies have revealed associations between microbiome community composition and host vaccine responses (recently reviewed in 152, 153). One common observation is that decreased vaccine response occurs in individuals with a disrupted microbiota (1). For example, several studies suggest that a normobiotic infant gut microbiome, replete with *Bifidobacterium*, promotes vaccine response, whereas a more dysbiotic microbiome harboring excess Proteobacteria interferes with vaccination outcome (154–159). Other studies have correspondingly shown that healthy human fecal material transplanted into neonatal gnotobiotic pigs or mice promotes strong responses to vaccines, while transplantation of dysbiotic human samples (harvested from individuals with intestinal enteropathy) dampens immune responses after vaccination (138, 160). Given the important role of the microbiota to educate the immune system during early development (161), it is possible that the dysbiotic infant microbiome could lead to immune deficiencies and reduced vaccine response. However, reducing the frequency of bacterial enteropathogens with azithromycin treatment prior to vaccination did not improve poliovirus vaccine response (162), suggesting that microbial dysbiosis may interfere with vaccine response *via* complex community changes or long-lasting immune effects.

Specific immunomodulatory bacteria have also been associated with vaccine response. In infants, two parallel studies found that a poor response to rotavirus vaccine was associated with increased *Bacteroides* and *Prevotella* spp., whereas a strong response was associated with higher levels of Proteobacteria and Firmicutes, particularly *Streptococcus bovis* (163, 164). Two additional studies have found that responders tend to have higher levels of Proteobacteria, though these observations were not statistically significant (165, 166). Nonetheless, a clinical study in adults found that vancomycin treatment, which recreates a similar microbial community comprising decreased Bacteroides and increased Proteobacteria, temporarily increased antibody levels in response to rotavirus (167). The authors speculate that in responders, highly immunostimulatory ligands such as LPS, peptidoglycan, or flagellin could promote viral infection or act as endogenous adjuvants to promote vaccine response.

Other immunomodulatory bacteria have been identified in studies of vaccine response in adults. A study with oral typhoid vaccine found that the gut abundance of the Firmicutes Lachnospiraceae and Ruminococcaceae was associated with early cell-mediated response to antigen after vaccination (168). Presence of the Firmicutes *Lactobacillus* and *Streptococcus*, as well the Bacteroidetes *Bacteroides* and *Prevotella*, in the nasal microbiome were associated with positive response to a nasal influenza vaccine (169). Furthermore, a recent meta-study analysis associated Actinobacteria and Firmicutes with positive vaccine responses, whereas Proteobacteria and Bacteroidetes were associated with poor vaccine outcomes (170). Though differences in study design, vaccine strategy, patient age, as well as other confounding variables complicates the interpretation of cross-study microbial associations, published studies together point toward an association of Firmicutes with successful vaccine outcome.

Interventional clinical studies including extensive immune characterization may facilitate more mechanistic explorations of the role of the microbiome during vaccination. A recent study in humans found that disruption of the microbiota by antibiotic treatment decreased response to influenza vaccination, specifically in individuals with low pre-existing immunity (171). Characterization of the metagenome and immune tone after antibiotic treatment implicated microbial bile acid metabolism, inflammasome signaling, and the underlying inflammatory state as key players that are influenced by the microbiota in the context of vaccination. We anticipate that similar investigational clinical studies may reveal crucial microbial and immune mechanisms that interact to either increase or decrease vaccine response in humans.

### Pre-Clinical Findings

Since the 1960s, germ-free mouse, chicken, and pig models have demonstrated that the microbiome influences vaccine response (119–124). More recently, microbiota disruption *via* antibiotic treatment in mice has corroborated these early observations (125–127, 130, 131, 133). Multiple antibiotics from different classes and with different breadths of activity have been shown to decrease vaccine response (**Table 3**). This observation suggests that diverse species may play a role in vaccine response. It also appears that the microbiome influences response to multiple classes of vaccines, including live, inactivated, and molecular vaccines (**Table 3**). Nearly all studies employing molecular or inactivated vaccines have found that microbiome disruption decreases response; in contrast, studies of live vaccines have found the microbiome can either promote, reduce, or have no effect on response (124, 126, 127, 130). These differences raise the possibility that complex interactions between the microbiome and the immune system may influence the ability of a live vaccine to colonize the host (colonization resistance), and thus have clear implications for considering the impact of the microbiome during development of live-attenuated (155, 168, 169) and viral- or bacterial-vectored vaccines (172, 173) and could also influence cellular transfection by mRNA-based vaccines.

Notably, several studies have identified specific microbial products or host factors that mediate the effects of the microbiome on vaccine response. Broadly expressed microbe-associated molecular patterns (MAMPs) may mediate key effects of the microbiota on vaccine response. Indeed, a recent study demonstrated that signaling by the pattern recognition receptor (PRR) TLR5, stimulated by the MAMP flagellin, is important for vaccine adjuvanticity (126). Depletion of the microbiota compromised parenteral vaccine response, and this defect could be reversed by colonization with flagellated bacteria but not by colonization with an aflagellated isogenic strain. Further, loss of TLR5 specifically in B cells compromised response to several unadjuvanted vaccines but did not affect response to adjuvanted vaccines. Since deletion of TLR5 did not alter baseline plasma cell phenotypes (126), the authors hypothesized that gut-derived flagellin spreads systemically and functions as an endogenous adjuvant at the site of immunization (174). However, another group was unable to reproduce the requirement for TLR5 for vaccine response (131), suggesting that differences in the baseline microbial community likely dictate the mechanisms by which the microbiome affects vaccine response. Nonetheless, the data suggest that particular microbial communities may function as an endogenous adjuvant, or that steady-state intestinal MAMP-PRR signaling can alter the systemic immunophenotype and alter response upon subsequent vaccination.

Indeed, altered homeostatic cytokine production in response to microbes has been implicated in vaccine response and protection from infection. One study found that induction of the immunosuppressive cytokine IL-10 by oral introduction of *Helicobacter hepaticus* (*Hh*) into immunocompetent mice disrupted response to intramuscular vaccination with live-attenuated *Mycobacterium tuberculosis* (143). The authors found that, compared to animals with a normal microbiota, *Hh*-colonized animals exhibited lower antigen-specific cell-mediated responses and higher infection when nasally challenged with *M. tuberculosis*. Infusion of IL-10-receptor-blocking antibodies restored vaccine response in *Hh*-colonized animals, demonstrating that the effect was mediated by increased IL-10 signaling. However, *Hh* colonization had no effect on IL-10 expression or Treg abundances in the lung but did increase IL-10 expression in the intestines, suggesting that intestinal IL-10 and other intermediate factors likely influence systemic immunity. Whether the effects of *Hh* on intestinal IL-10 production are direct as observed for *Hh* and T cells (175), or are due to *Hh*-mediated microbiome disruption (176) remains unknown. A second study also found that colonization of mice with viruses and a helminth disrupted response to vaccination with live-attenuated yellow fever virus, and observed that at the time of vaccination, colonized animals displayed altered expression of several inflammatory cytokines which could compromise vaccine response (144). Thus, microbially-induced changes in the steady-state cytokine milieu likely affect subsequent systemic immunity to vaccines.

Other studies suggest the microbiota are required to supply immune receptor ligands for the activity of mucosal adjuvants. Two independent groups have employed immunization with albumin and cholera toxin (CTx) to demonstrate that germ-free or antibiotic-treated mice have decreased antibody responses, which are rescued by supplementation with microbial molecules (128, 129, 132). Both groups demonstrated that CTx potentiates immune receptor signaling, which require the presence of microbial molecules for full signaling activation in response to CTx. One study found that microbial peptidoglycan signaled through NOD2 during vaccination with CTx, leading to increased IL-1β production and generation of T-follicular helper cells and plasma cells (128, 129). A second group found that microbe-derived short-chain fatty acids (SCFAs) synergized with CT to promote vaccine response, by driving DC-mediated production of B cell activators BAFF and retinoic acid (132). Notably, the authors also found that SCFAs increased vaccine response even in the absence of CTx through the same immune pathway, demonstrating that SCFAs can independently promote pro-vaccine immune tone, in addition to mediating the adjuvant effect of CTx. It is important to note that the two groups employed different antibiotic cocktails, which likely differentially affect the microbiome, which may account for the distinct mechanisms identified.

Taken together, these preclinical studies reveal several possible mechanisms by which the microbiota can promote immune response in the context of vaccine response: as an endogenous adjuvant, as ligands for immune receptors that are potentiated by exogenous adjuvants, and as regulators of systemic immune tone. Germ-free and antibiotic-treated mice represent crucial models to parse the mechanisms by which the microbiome affects vaccine response. Another relevant model for future studies is “dirty” mice, which receive microbes from pet store mice *via* cohousing or bedding transfer, and may prove a valuable system for understanding vaccine response (177). Given that there are multiple mechanisms by which the microbiome can affect vaccine response and that these effects can vary across different vaccines, additional preclinical studies are critically important to define the microbes and immune pathways involved.

### The Microbiome Modulates Vaccine Response in Infants and in the Elderly

Though infants and the elderly are profoundly at risk for severe infection, these populations also consistently fail to respond to vaccines (reviewed in 178–180). Lack of vaccine response can be attributed to maternal antibody interference (reviewed in 181, 182), early life immune immaturity (183–185), and immunosenescence in the elderly (186–192). However, emerging evidence suggests that microbiota may also influence vaccine response at the extremes of life, likely by modulating immune development and senescence (153).

The microbiome plays a key role in the proper maturation of the immune system (reviewed in 161), which is necessary for optimal vaccine response. Alterations in the neonatal microbiome are associated with defects in vaccine response in clinical studies (154, 163, 164) and in preclinical studies (125, 131). Although the mechanisms influencing vaccine response in neonates have not been clearly defined, related studies suggest that early-life microbial stimulation could promote vaccine response indirectly by modulating immune development and function (193–195), or directly by regulating adjuvanticity during vaccination (126). The possibility also exists that microbial dysbiosis during early life could interfere with vaccine response later in life, due to defects in microbial-immune imprinting (reviewed in 196), as has been observed for other immune-related disorders (9, 14, 15, 197). Although this idea has not yet been examined in clinical studies of vaccine response, a study in mice found that early life microbial dysfunction did not affect later vaccine response, provided the microbiome was repaired prior to vaccination (131). Thus, further studies are warranted to explore the immediate and long-term ramifications of early-life microbial dysbiosis on vaccine responses and relevant immune pathways; such discoveries could reveal novel therapeutic strategies to improve vaccine response in neonates by harnessing the microbiota.

In the elderly, emerging evidence suggests that age-induced changes in the microbiome contribute to immunosenescence (145, 198–200) and could thereby mediate changes in vaccine response (201). Indeed, defective intestinal germinal center reactions in aged mice were rescued by transplantation with fecal material from young mice (145), which could suggest an improved capacity for vaccine response after fecal transplantation. In humans, immunophenotypic similarities have been observed between elderly subjects and adults with antibiotic-induced microbial dysbiosis, consistent with the idea that age-mediated effects and antibiotic-mediated effects on vaccine response are both effected by the microbiota (171, 192). Others have speculated that immunosenescence caused by changes in the microbiota could drive other pathological immune conditions such as asthma (202). Thus, in both elderly and neonatal individuals, the microbiome may mediate changes in vaccine response, and microbiome rehabilitation represents a promising approach to promote vaccine response in these populations.

### Microbial Manipulation to Improve Vaccine Response

Given the role of the microbiome in vaccine response in clinical and preclinical studies, a key question is whether manipulating the microbiome can improve vaccine outcome, especially in populations at risk for poor vaccine response (182). Many clinical studies have evaluated vaccine response after dietary supplementation with probiotics, primarily species within *Lactobacillus* or *Bifidobacterium*. Recent reviews and meta-analyses of these studies highlight that there is significant heterogeneity in the ability of probiotic supplementation to increase antibody titers after vaccination (203–206). Effects are likely to be specific to particular bacterial strains, and may vary between different vaccines and adjuvants (149, 207). Several of these probiotic studies have also found correlates between probiotic supplementation and diverse measures of immune function, including serum levels of pro-inflammatory cytokines (208, 209), T cell responsiveness (210, 211), and innate immune cell activity (212, 213), although the mechanistic implications of these observations are unclear.

The effect of probiotics on vaccine response has also been evaluated in neonatal pigs, and recent studies have begun to elucidate the mechanisms by which *Lactobacillus* and *Bifidobacterium* strains promote vaccine response in this model. Early studies using gnotobiotic pigs revealed that colonization by probiotics prior to oral rotavirus immunization enhanced Th1 cellular immunity (139–141). However, prior colonization by human fecal material prevented subsequent *Lactobacillus* colonization and *Lactobacillus*-mediated effects on vaccine response (142), suggesting that an intact microbiota can obstruct the effects of probiotics and contribute to the heterogeneity of their effects across individuals.

Efforts are underway to use probiotic strains as vaccine vectors to capitalize on the vaccine-promoting and immunomodulatory effects of certain microbes, by engineering the expression of antigens to induce the desired immune responses including mucosal IgA (reviewed in 214, 215). In particular, Lactobacilli are known to activate the immune cell receptors NOD2, TLR2, TLR6, C-type lectin receptors, and the caspase-1 dependent inflammasome (reviewed in 216). Recently, a study demonstrated that activation of NOD2 was required for Th2 skewing and humoral immune responses to a *Lactobacillus* vaccine vector strain (146). Further work to elucidate the cell types and functional pathways modulated by *Lactobacillus* and other probiotics will contribute to understanding of key interactions between the microbiota and the immune system in the context of vaccines.

Several groups have also explored whether prebiotic supplementation improves vaccine outcome. Prebiotics are expected to promote a diverse microbiota and prevent expansion of dysbiosis-inducing microbes (149). Though animal studies suggest prebiotics can stabilize “normobiotic” microbes including *Lactobacillus* and *Bifidobacterium* and promote vaccine response (134–138), prebiotics in clinical studies have predominantly had no effect on vaccine outcome (217–222). Why prebiotics do not affect vaccine response remains unclear, as they have been shown to influence other immune-related conditions (reviewed in 149).

Taken together, these and previously discussed studies illustrate the potential of microbiome-modulating interventions to promote vaccine response, but suggest that effective realization of such strategies is challenging. We note that the majority of studies have sought to enhance the abundance of Lactobacilli or Bifidobacteria, which are known to modulate the immune system (216, 223). These families belong respectively to the Firmicutes and Actinobacteria, which have been associated with vaccine response in a meta-analysis of clinical studies (170). It is possible that vaccine response could also be augmented by probiotic supplementation with other families within the Firmicutes, though this possibility has not been widely investigated. An outstanding question for probiotic approaches is whether it is necessary for probiotic strains to colonize within the context of an intact microbiome in order to promote vaccine response. Finally, an alternative strategy may be to leverage the pathways modulated by microbiome, either by delivery of live engineered organisms (146) or of bioactive molecules (132).

## Concluding Remarks: Interactions Between Microbiome and the Immune System have the Potential to Shape Response to Immune Interventions

**Figure 2:**
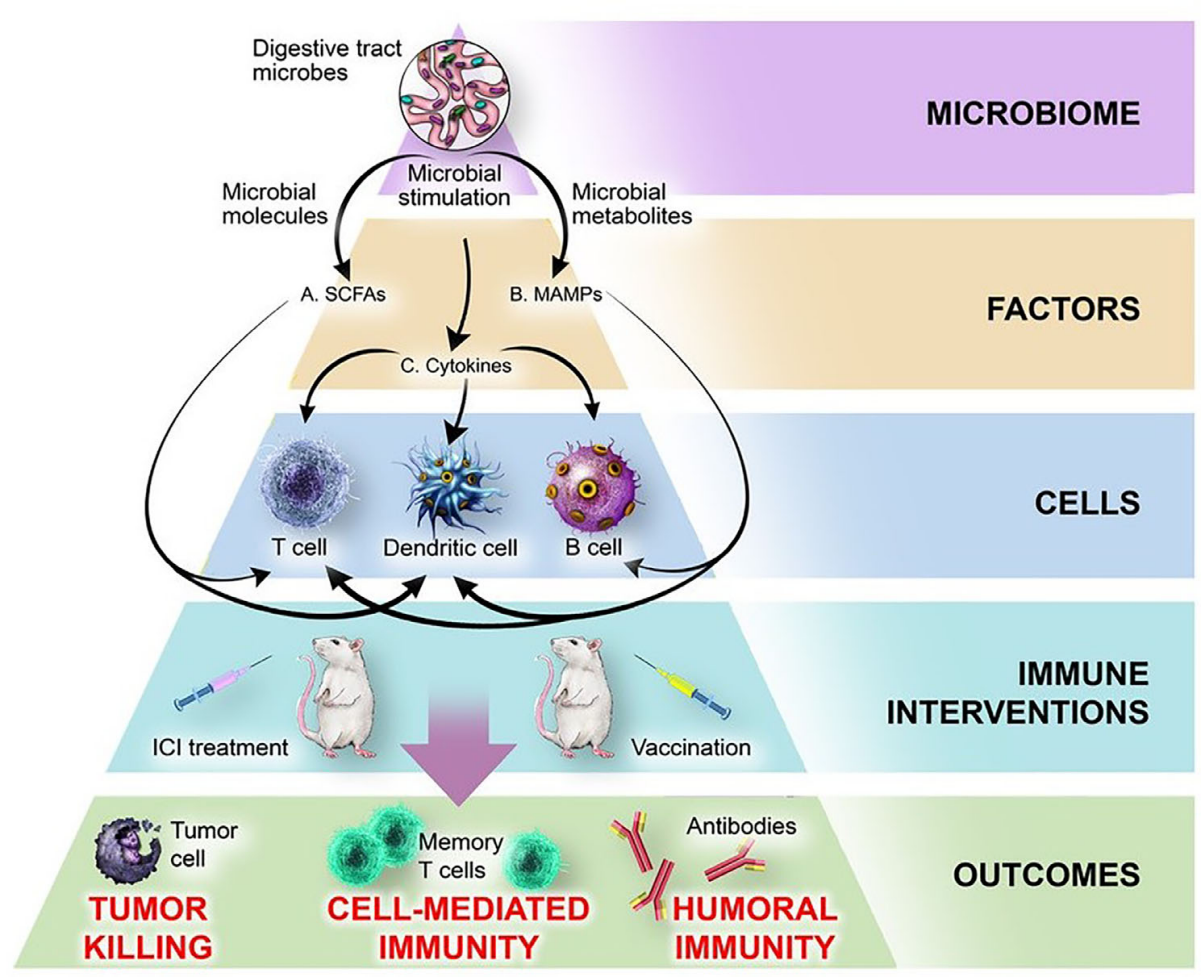
Mechanisms by which the microbiome influences response to ICI treatment and vaccination. The microbiome produces microbial factors and influences host factors and cells, thereby influencing the outcomes of immune interventions. **(A)** Microbiome-derived short-chain fatty acids (SCFAs) bind receptors including GPR43 on DCs and T cells, leading to changes in cytokine production, antigen presentation, cellular polarization, and interactions with other cell types. **(B)** Microbe-associated molecular patterns (MAMPs) including flagellin, polysaccharide A, fucosylated antigens, unmethylated CpG DNA, and peptidoglycan bind pattern recognition receptors on DCs (NOD2, TLR2, TLR3, TLR9, DC-SIGN) or B cells (TLR5) and modulate activation, cytokine production, and immune cell function. **(C)** Microbiome-dependent changes in production of cytokines (IL-1β, IL-12, IL-18, IFN-γ, IL-10) produced by intestinal DCs or epithelial cells broadly affect immune cell function.

Recent literature reveals that assorted microbes, metabolites, and immune factors interact to influence the patient response during cancer immunotherapy and immunization (summarized in **Tables 1** **–** **3**). To gain greater perspective into the most meaningful molecular and cellular mechanisms by which the microbiome modulates the immune system, we have juxtaposed two therapeutic spheres: ICIs and vaccines. Evidence from both therapeutic spheres highlights three elements of the microbiome that consistently play an immunomodulatory role: microbially-derived metabolites including short-chain fatty acids, microbe-associated molecular patterns (MAMPs), and inflammatory cytokines (**Figure 2**).

**Table 1:** Gut microbial composition is associated with the efficacy of immune checkpoint inhibitor therapy in patients.

Cancer Type (number of patients)	Immune checkpoint inhibitor	Identified factor	Key associations	Reference
Metastatic non-small-cell lung carcinoma (74)	anti-PD-1	Antibiotic prescription (within 3 months prior)	No association with progression-free survival	70
Metastatic renal cell (121) and non-small-cell lung (239) carcinomas	anti-PD-L1	Antibiotic usage (within 30 days prior)	Reduced ICI response^a^	65
Metastatic renal cell (67), non-small-cell lung (140), and urothelial carcinoma (42)	anti-PD-1 or anti-PD-L1 anti-PD-1 or anti-PD-L1	Bacteria: *Akkermansia muciniphilia*	Enhanced ICI response^a^	58
Metastatic melanoma (26)	anti-CTLA-4	Bacteria: *Faecalibacterium* spp. and Firmicutes	Longer progression-free survival	59
Metastatic melanoma (39)	anti-CTLA-4	Bacteria: *Faecalibacterium prausnitzii*, *Bacteroides thetaioamicron*, *Holdemania filiformis*	Enhanced ICI response^a^	60
	anti-PD-1	Bacteria: *Dorea formicigenerans*	Enhanced ICI response^a^	
	anti-CTLA-4 or anti-PD-1	Xenobiotic: anacardic acid	Enhanced ICI response^a^	
Metastatic melanoma (43)	anti-PD-1	Bacteria: *Faecalibacterium prausnitzii; Ruminococcus bromii*; microbial diversity baseline	Enhanced ICI response^a^	61
Metastatic melanoma (42)	anti-PD-1; anti-CTLA-4	Bacteria: *Bifidobacterium longum*; *Collinsella aerofaciens*; *Enterococcus faecium*	Enhanced ICI response^a^	62
Metastatic melanoma (27)	anti-PD-1; anti-CTLA-4	Bacteria: Microbial community richness	Longer progression-free survival	64
Metastatic non-small-cell lung carcinoma (142)	anti-PD-1; anti-PD-L1	Antibiotic treatment (concomitant)	Shorter progression-free survival and overall survival	67
Metastatic renal cell carcinoma (69)	anti-PD-1	Antibiotic usage (within 2 months prior)	Shorter progression-free survival	66
Metastatic melanoma (568)	anti-PD-1; anti-CTLA-4	Antibiotic usage (within 3 months prior)	Shorter overall survival	68
Metastatic non-small-cell lung carcinoma (2208)	anti-PD-1; anti-CTLA-4	Antibiotic usage (within 3 months prior or concomitant)	Shorter median overall survival	69
Metastatic melanoma (50)	anti-CTLA-4	Bacteria: *Faecalibacterium* spp.; *Gemminger spp*	Longer progression-free survival	63
		Metabolite: fecal SCFA butyrate	Shorter progression-free survival	
Metastatic non-small cell lung carcinoma	anti-PD1; anti-PD-1	Bacterial delivery: *Clostridium butyricus*	Longer progression-free survival	71

a Response determined by biomarker-based disease progression criteria.

**Table 2:** Impact of gut microbiome on immune checkpoint inhibitor therapy: selected preclinical studies.

Tumor Cell Model (Cancer Type)^a^	Treatment	Immune checkpoint inhibitor	Key findings	Reference
B16.SIY (M)	Comparison of mice from different vendors and different microbial communities	anti-PD-L1	Differential tumor growth in mice from different vendors; Bifidobacterium promotes antitumor immunity and anti-PD-L1 efficacy	82
MCA-205 (FS) RET (M) MC38 (CRC)	Antibiotic cocktail: ampicillin, streptomycin, & colistin	anti-CTLA-4	*Bacteroides fragilis* promotes anti-CTLA-4 efficacy *via* TL2/TLR4 host signaling	83
RET (M) RENCA (RCC) MCA-205 (FS)	Antibiotic cocktail: ampicillin, streptomycin, & colistin	anti-PD-1 ± anti-CTLA-4	Antibiotic exposure decreased ICI efficacy; oral supplementation with *Akkermansia muciniphila* restored the efficacy of ICI	58
MC38 (CRC) *Braf* ^V600E^ *Pten* ^−/−^ (mouse-derived melanoma)	Colonization with a consortium of 11 fecal strains	anti-PD-1 ± anti-CTLA-4	Colonization with an 11-strain consortium induces IFN-γ producing CD8^+^ T cells and increases ICI efficacy	84
CT26 (CRC)	Antibiotic: ampicillin, streptomycin, & colistin (cocktail); vancomycin; colistin	anti-PD-1	Antibiotics decreased efficacy of anti-PD-1 therapy and altered glycerophosphlipid metabolism	85
MC38 (CRC) CT26 (CRC)	Prebiotic supplementation: butyrate	anti-CTLA-4	SCFA butyrate supplementation reduces efficacy of anti-CTLA-4	63
MC38 (CRC) MB49 (RCC) B16-F10 (M) Genetic CRC (Msh2)	Colonization with *Bifidobacterium pseudolongum*; delivery of inosine and microbial ligands	anti-CTLA-4	Microbial-derived inosine activates anti-tumor T cell *via* the adenosine A_2A_ receptor in combination with T cell costimulation by MAMPs	86

a M, melanoma; FS, fibrosarcoma; CRC, colorectal carcinoma; RCC, renal cell carcinoma.

**Table 3:** Summary of preclinical studies linking microbiome and vaccine outcome.

Vaccine (route^a^)	Model/Treatment	Key Findings	Reference
**Studies in Germ-Free Models**			
Bovine gamma-globulin (SC)	Germ-free mice	Reduced serum IgG antibody response	119
*E coli* O antigen (PO)	Germ-free pigs	Reduced IgA-positive cells in lamina propria	120
Sheep red blood cells (IP); Bovine serum albumin (IP)	Germ-free mice	Reduced serum IgG antibody response	121
Heat-inactivated *E. coli* (PO)	Germ-free chickens	Reduced intestinal and serum antibody (IgG, IgA) production	122
Sheep red blood cells (SC)	Germ-free mice	Reduced delayed-type hypersensitivity response; microbiota restoration restored response	123
Live attenuated Bacille Calmette–Guerin (IV)	Germ-free mice	Enhanced resistance to *Mycobacterium tuberculosis* challenge after immunization	124
Ovalbumin + complete Freund’s adjuvant (SC)	Germ-free mice	Reduced ova-specific antibody response	125
Trivalent inactivated influenza (SC)	Germ-free mice	Reduced antigen-specific serum IgG	126
Attenuated human rotavirus (PO)	Germ-free mice	Enhanced antigen-specific antibody response	127
Human serum albumin + cholera toxin (PO or IN)	Germ-free mice	Reduced ova-specific plasma IgG	128
Human serum albumin + cholera toxin (PO)	Germ-free mice	Reduced ova-specific plasma IgG	129
**Studies of Antibiotic Treatment**			
Tetanus toxoid + alum (SC) Pneumococcal polysaccharides (SC) Hepatitis B surface antigen + alum (IP) Live-attenuated *S. typhi* Ty21A (IP)	Antibiotics in mice: Clarithromycin or doxycycline (4 weeks)	Reduced vaccine-specific serum IgM antibody levels	130
Ovalbumin + complete Freund’s adjuvant (SC)	Antibiotic cocktail in mice: clindamycin, ampicillin, & streptomycin (maternal 5 days)	Reduced ova-specific antibody response in pups from antibiotic-treated dams	125
Live attenuated human rotavirus (PO)	Antibiotic cocktail in mice: Ampicillin & Neomycin (2 weeks)	Enhanced antigen-specific antibody response	127
Trivalent inactivated influenza (SC)^b^	Antibiotics in mice (4 weeks): cocktail of neomycin, ampicillin, Vancomycin, & metronidazole; vancomycin; polymixin B	Reduced antigen-specific serum IgG	126
Tetanus toxoid + diphtheria toxoid + acellular pertussis antigens + alum (SC) HIV-gp140 + alum (SC) Live attenuated yellow fever YF-17D (SC)	Antibiotics in mice (4 weeks): cocktail of neomycin, ampicillin, Vancomycin, & metronidazole	No effect on antigen-specific IgG	126
Human serum albumin + cholera toxin (PO; IN)	Antibiotic cocktail in mice: ampicillin, vancomycin, metronidazole, neomycin (3-4 weeks)	Reduced ova-specific plasma IgG	128
Live attenuated Bacille Calmette-Guerin (SC) Pneumococcal polysaccharide-diphtheria toxoid conjugate + alum (IP) Meningococcal B surface proteins + outer membrane vesicles (IP) Meningococcal C polysaccharide-tetanus toxoid conjugate + alum (IP) Diphtheria toxoid + tetanus toxoid + pertussis toxoid + pertussis proteins + hepatitis B surface antigen + inactivated polioviruses + *Haemophilus influenzae* type b-polysaccharide + alum (IP) Trivalent inactivated influenza (SC)^b^	Antibiotic cocktail in mice: Ampicillin & neomycin (maternal 2-5 weeks)	Reduced vaccine-specific IgG titer	131
Live attenuated Bacille Calmette-Guerin (SC) Pneumococcal polysaccharide-diphtheria toxoid conjugate + alum (IP)	Antibiotic cocktail in mice: ampicillin & neomycin (3 weeks)	No effect on vaccine-specific IgG titer	131
Ovalbumin + cholera toxin (PO)	Antibiotic cocktail in mice: Metronidazole, vancomycin, ampicillin, kanamycin (10 days)	Reduced ova-specific fecal IgA and serum IgG	132
Rabies vaccine iLBNSE (IM)	Antibiotic cocktail in mice: metronidazole, vancomycin, ampicillin, neomycin (4 weeks)	Reduced rabies-specific IgG, IgM, neutralizing antibodies; reduced Tfh cells, germinal center B cells, memory response	133
**Studies of Prebiotic Supplementation**			
Trivalent inactivated influenza (SC)	Prebiotic cocktail in mice: galacto- and fructo-oligosaccharides	Enhanced delayed-type hypersensitivity response; increased levels of Bifidobacteria and Lactobacilli	134
Trivalent inactivated influenza (SC)	Prebiotic cocktail in mice: fructo-oligosaccharides and inulin	No effect on delayed-type hypersensitivity response; increased levels of Bifidobacteria and Lactobacilli	134
Live attenuated *Salmonella typhimurium* (oral)	Prebiotic cocktail in mice: fructo-oligosaccharides and inulin	Enhanced antigen-specific antibody titer, inflammatory cytokines, and survival after pathogen challenge	135
Trivalent inactivated influenza (SC)	Prebiotic cocktail in mice: Galacto- and fructo-oligosaccharides	Enhanced delayed-type hypersensitivity response; increased levels of Bifidobacteria and Lactobacilli	136
Ovalbumin + cholera toxin (oral)	Prebiotic cocktail: acetate and butyrate	Enhanced vaccine response and production of B-cell-activating factors in dendritic cells; **effect was dependent on SCFA-receptor GPR43**	132
Trivalent inactivated influenza (SC)	Prebiotic cocktail in mice: Galacto- and fructo-oligosaccharides 2’FL	Enhanced antigen-specific antibody titer and IL-6 production in male mice, increased levels of Actinobacteria	137
Cholera toxin + ovalbumin (oral)	Prebiotic cocktail: spirulina, amaranth, flaxseed, micronutrients	Enhanced antigen-specific antibody titer and germinal B cell frequency in mesenteric lymph nodes; effect was dependent on presence of particular microbes	138
**Studies of Microbial Delivery**			
Ovalbumin + complete Freund’s adjuvant (SC)	Conventionalization of germ-free mice	Enhanced ova-specific antibody response	125
Live attenuated human rotavirus (PO)	Probiotic in gnotobiotic neonatal pigs: *Lactobacillus acidophilus*	Modulated balance of antigen-specific Th1 cells and Tregs in a dose-specific manner	139
Live attenuated human rotavirus (PO)	Probiotic cocktail in gnotobiotic neonatal pigs: *Lactobacillus rhamnosus* GG and *Bifidobacterium animalis lactis* Bb12	Enhanced antigen-specific Th1 response and protection from rotavirus challenge	140
Live attenuated human rotavirus (PO)	Probiotic cocktail in gnotobiotic neonatal pigs: *Lactobacillus rhamnosus* GG and *Bifidobacterium animalis lactis* Bb12	Enhanced intestinal antigen-specific antibody titers, cell responses, and protection from rotavirus challenge	141
Live attenuated human rotavirus (PO)	Probiotic in neonatal gnotobiotic pigs pre-colonized with human fecal material: *Lactobacillus rhamnosus GG*	No effect on protection from rotavirus challenge; modulated production of antigen-specific Th1 cells in a dose- and colonization-dependent manner	142
Trivalent inactivated influenza (SC)	Single strain in germ-free mice: flagellated or aflagellated *E. coli* Conventionalization of germ-free mice	Enhanced antigen-specific antibody response after conventionalization or colonization with flagellated, but not aflagellated, bacteria **MAMP flagellin functions as an endogenous adjuvant**	126
Live attenuated *Mycobacterium tuberculosis* Ad85A (IM)	Pathobiont in mice: *Helicobacter hepaticus* colonization	Reduced protection from *Mycobacterium tuberculosis* **IL10 production reduces vaccine response**	143
Live attenuated yellow fever virus YFV-17D (SC)	Pathobiont in mice: MHV86, MCMV, influenza WSN, & *Heligmosomoides polygyrus*	**Reduced pre-immunization production of inflammatory cytokines correlates with reduced anti-YFV antibody response**	144
Pneumococcal polysaccharide-diphtheria toxoid conjugate + alum (IP)	FMT in antibiotic-treated mice: fecal material from untreated mice	Enhanced vaccine-specific antibody titer	131
Keyhole limpet hemocyanin + alum (SC); NP conjugated to cholera toxin (PO)	Co-housing of aged mice with young mice FMT in aged mice: fecal material from young mice	No effect on antibody responses; improved germinal center reactions independent of vaccination	145
Cholera toxin + ovalbumin (PO)	FMT in germ-free mice: fecal material from undernourished children Co-housing of mice that received different FMT Consortium of five fecal strains*: Bacteroides acidifaciens, Bacteroides fragilis, Clostridioides difficile, Costridium innocuum, Fusobacterium mortiferum*	**Specific microbes mediate the pro-vaccine effects of prebiotics**	138
**Studies in Genetically Engineered Host Models**			
Trivalent influenza (SC)^b^ Inactivated poliovirus (SC)	TLR5^−/−^ mice	Reduced antigen-specific serum IgG for nonadjuvanted vaccines **TLR5 mediates endogenous adjuvant response to vaccine**	126
Live attenuated yellow fever YF-17D (SC) Hepatitis B sAg + alum (SC)	TLR5^−/−^ mice	No effect on antigen-specific serum IgG	126
Human serum albumin + cholera toxin (PO; IN)	Nod2^−/−^ mice Nod2-DC(CD11c) specific deletion	Reduced ova-specific plasma IgG **Nod2 in DCs mediates adjuvant activity of cholera toxin**	128
Human serum albumin + cholera toxin (PO; IN)	Myd88^−/−^; Ripk2^−/−^; Nod1^−/−^	No effect on ova-specific plasma IgG	128
*Lactobacillus acidophilus* vaccine strain expressing HIV proteins (PO)	Nod2^−/−^ mice	Reduced antigen-specific IgG and IgA **Nod2 mediates response to vaccine strain**	146
Trivalent influenza (SC)^b^	TLR5^−/−^ mice	No effect on antigen-specific serum IgG	131
Human serum albumin + cholera toxin (PO)	Nod2^−/−^ mice IL1b^−/−^ mice	Reduced ova-specific plasma IgG **IL-1β production *via* Nod2 is required for cholera toxin adjuvanticity**	129
Ovalbumin + cholera toxin (PO)	GPR43^−/−^ mice	Reduced ova-specific fecal IgA and serum IgG; **reduced pro-vaccine effect of SCFA prebiotics** **GPR43 mediates pro-vaccine effect of SCFAs**	132

^a^SC, subcutaneous; IP, intraperitoneal; PO, oral; IV, intravenous; IN, intranasal; IM, intramuscular; ^b^Noted discrepancies with regard to effect of antibiotics and TLR5^−/−^ on response to influenza vaccine could be attributed to different underlying microbial communities across study location; Key mechanistic insights are noted in **bold text.**

The microbiome is a rich source of diverse bioactive metabolites that can affect host biology and the immune system (reviewed in 150, 224, 225). One class of bacterial metabolites that influence response to both vaccination and ICI therapy are short-chain fatty acids (SCFAs). SCFAs are immunomodulatory metabolites and are produced by a subset of intestinal microbes (reviewed in 224, 226), some of which are implicated in clinical studies of immunotherapy and vaccine response. SCFAs such as butyrate and propionate appear to alter immunotherapy outcome by modulating CTL activation directly or indirectly by influencing DC maturation (37, 63, 227), whereas butyrate, acetate and some branched-chain fatty acids may alter vaccine response by modulating DC-mediated B cell activation (132, 228, 229). SCFAs are known to broadly influence the immune system as well as other host pathways, and have also been implicated in regulation of Tregs, allergic disease, resistance to infectious disease, obesity, intestinal barrier function, carcinogenesis, and microglia development in the central nervous system (40, 224, 226, 230–235). However, future investigations must continue to define the biology of SCFAs, as well as other immunomodulatory microbial metabolites associated with outcomes of immune-targeted interventions (84, 85), including whether immunotherapies themselves influence the gut metabolome. Determining the unique effects of specific metabolites, including SCFAs, on immune pathways critical for ICI and vaccine response may identify key microbes and signaling pathways that could be harnessed to promote therapeutic success.

The outcomes of ICI and immunization are also influenced by MAMPs, including flagellin, unmethylated CpG DNA, peptidoglycan, muramyl dipeptide, and polysaccharides (6, 83, 126, 129). MAMPs directly and indirectly modulate activation and differentiation of immune cells (236–238), and synthetic MAMPs are already used as exogenous adjuvants during immunization to activate the immune system. In the context of microbial modulation of immunotherapy and vaccination outcomes, the cellular and molecular mechanisms by which commensal MAMPs affect B and T cells are incompletely defined. Commensal MAMPs can bind to host receptors and directly influence B cells during vaccination (126), possibly by promoting antibody production and regulating class-switching (239, 240). Similarly, endogenous MAMPs may also promote T cell function as costimulatory molecules (241–245). B and T cell functions are also indirectly influenced by MAMP-mediated modulation of APC maturation, leading to altered interactions and production of cytokines and chemokines (5, 6, 83, 129, 246, 247). By understanding the role endogenous MAMPs play in outcomes of ICI and vaccination, we may be able to identify key MAMPs and host pathways that promote immunotherapy response, which could lead to development of novel vaccine adjuvants or ICI co-therapies.

Inflammatory cytokines are emerging as a key host mediator of microbial immunomodulation (54). Both ICI and vaccine outcomes have been associated with microbial modulation of inflammatory cytokines, including IL-12, IL-1β, and IFN-γ (6, 58, 83, 85, 129). These effects appear to be primarily mediated by MAMP stimulation of host receptors on APCs, but stimulation of epithelial cells with MAMPs or microbial metabolites can also alter epithelial cytokine production and impact immunity (143, 173, 248, 249). As the core communication toolbox of the immune system, cytokines represent a likely mediator through which the microbiome and intestinal immunity could influence systemic responses during ICI treatment and vaccination (58, 143, 247). Thus, microbiome-derived products could represent an indirect but powerful approach to control cytokine levels and broadly affect the immune system in diverse therapeutic contexts.

The effect of the microbiome on the outcome of immunotherapies certainly extends beyond ICI therapy and vaccination, as has been observed for non-ICI immunostimulatory treatments to control tumor growth (25, 26), anti-inflammatory treatment for irritable bowel syndrome and rheumatoid arthritis (27–30), and antigen desensitization for allergy (31). It is also clear that the microbiome broadly affects the immune system, as has been well-documented in studies of infectious disease and immune function (5, 6, 230, 232, 235, 246, 247). Taken as a whole, the current literature argues that the microbiome likely plays a key role in the heterogeneity of the immune response across individuals. Though the majority of mechanistic studies to date have employed preclinical models, technological developments for *ex vivo* microbiome studies (58, 61, 138, 160), and microbiome manipulation of human subjects in the clinical setting (162, 167, 171) will facilitate our understanding of the role of the microbiota on the human immune system. As crucial immunomodulatory interactions continue to be identified, novel strategies also need to be developed to manipulate the microbiota and microbial regulation of immunity. Patient microbial profiles could be surveyed to anticipate therapeutic outcomes. Probiotics and prebiotics may play a role in repairing or supporting the microbiota (84, 138). Supplementation with immunomodulatory microbial metabolites or ligands (132), or direct targeting of microbially-regulated immune pathways (6, 247) could also bypass the effects of the microbiota on cytokine production and cellular function. As we develop novel approaches to understand and influence the microbiome, we will also expand our ability to harness the immune system to treat or prevent disease.

## Author Contributions

ALR, DJC, XL, JEN, and GS designed the work, collected and interpreted data, and approved the final work. ALR, DJC, and XL drafted the manuscript. GS, AGT, and DJH critically reviewed and approved the final work. All authors contributed to the article and approved the submitted version.

## Conflict of Interest

All authors are employees of Merck Sharp & Dohme Corp., a subsidiary of Merck & Co., Inc., Kenilworth, NJ, USA and may hold stock in Merck & Co., Inc. Kenilworth, NJ, USA.

## References

[1] Human Microbiome Project C. Structure, Function and Diversity of the Healthy Human Microbiome. Nature (2012) 486(7402):207–14. 10.1038/nature11234 PMC3564958 22699609

[2] Hollister EB Gao C Versalovic J. Compositional and Functional Features of the Gastrointestinal Microbiome and Their Effects on Human Health. Gastroenterology (2014) 146(6):1449–58. 10.1053/j.gastro.2014.01.052 PMC4181834 24486050

[3] Ursell LK Haiser HJ Van Treuren W Garg N Reddivari L Vanamala J. The Intestinal Metabolome: An Intersection Between Microbiota and Host. Gastroenterology (2014) 146(6):1470–6. 10.1053/j.gastro.2014.03.001 PMC4102302 24631493

[4] Zheng D Liwinski T Elinav E. Interaction Between Microbiota and Immunity in Health and Disease. Cell Res (2020) 30(6):492–506. 10.1038/s41422-020-0332-7 32433595 PMC7264227

[5] Ramakrishna C Kujawski M Chu H Li L Mazmanian SK Cantin EM. Bacteroides Fragilis Polysaccharide A Induces IL-10 Secreting B and T Cells That Prevent Viral Encephalitis. Nat Commun (2019) 10(1):2153. 10.1038/s41467-019-09884-6 31089128 PMC6517419

[6] Ichinohe T Pang IK Kumamoto Y Peaper DR Ho JH Murray TS. Microbiota Regulates Immune Defense Against Respiratory Tract Influenza A Virus Infection. Proc Natl Acad Sci USA (2011) 108(13):5354–9. 10.1073/pnas.1019378108 PMC3069176 21402903

[7] Thackray LB Handley SA Gorman MJ Poddar S Bagadia P Briseno CG. Oral Antibiotic Treatment of Mice Exacerbates the Disease Severity of Multiple Flavivirus Infections. Cell Rep (2018) 22(13):3440–53.e6. 10.1016/j.celrep.2018.03.001 29590614 PMC5908250

[8] Hapfelmeier S Lawson MA Slack E Kirundi JK Stoel M Heikenwalder M. Reversible Microbial Colonization of Germ-Free Mice Reveals the Dynamics of IgA Immune Responses. Science (2010) 328(5986):1705–9. 10.1126/science.1188454 PMC3923373 20576892

[9] Cahenzli J Koller Y Wyss M Geuking MB McCoy KD. Intestinal Microbial Diversity During Early-Life Colonization Shapes Long-Term IgE Levels. Cell Host Microbe (2013) 14(5):559–70. 10.1016/j.chom.2013.10.004 PMC4049278 24237701

[10] Seo SU Kamada N Munoz-Planillo R Kim YG Kim D Koizumi Y. Distinct Commensals Induce Interleukin-1beta Via NLRP3 Inflammasome in Inflammatory Monocytes to Promote Intestinal Inflammation in Response to Injury. Immunity (2015) 42(4):744–55. 10.1016/j.immuni.2015.03.004 PMC4408263 25862092

[11] Mazmanian SK Round JL Kasper DL. A Microbial Symbiosis Factor Prevents Intestinal Inflammatory Disease. Nature (2008) 453(7195):620–5. 10.1038/nature07008 18509436

[12] Castro-Dopico T Dennison TW Ferdinand JR Mathews RJ Fleming A Clift D. Anti-Commensal IgG Drives Intestinal Inflammation and Type 17 Immunity in Ulcerative Colitis. Immunity (2019) 50(4):1099–114.e10. 10.1016/j.immuni.2019.02.006 30876876 PMC6477154

[13] Teng F Klinger CN Felix KM Bradley CP Wu E Tran NL. Gut Microbiota Drive Autoimmune Arthritis by Promoting Differentiation and Migration of Peyer’s Patch T Follicular Helper Cells. Immunity (2016) 44(4):875–88. 10.1016/j.immuni.2016.03.013 PMC5296410 27096318

[14] Vatanen T Kostic AD d’Hennezel E Siljander H Franzosa EA Yassour M. Variation in Microbiome Lps Immunogenicity Contributes to Autoimmunity in Humans. Cell (2016) 165(6):1551. 10.1016/j.cell.2016.05.056 27259157

[15] Wang M Karlsson C Olsson C Adlerberth I Wold AE Strachan DP. Reduced Diversity in the Early Fecal Microbiota of Infants With Atopic Eczema. J Allergy Clin Immunol (2008) 121(1):129–34. 10.1016/j.jaci.2007.09.011 18028995

[16] Gomez de Aguero M Ganal-Vonarburg SC Fuhrer T Rupp S Uchimura Y Li H. The Maternal Microbiota Drives Early Postnatal Innate Immune Development. Science (2016) 351(6279):1296–302. 10.1126/science.aad2571 26989247

[17] Wu K Yuan Y Yu H Dai X Wang S Sun Z. Gut Microbial Metabolite Trimethylamine N-oxide Aggravates GVHD by Inducing M1 Macrophage Polarization in Mice. Blood (2020) 136(4):501–15. 10.1182/blood.2019003990 PMC7378459 32291445

[18] Schulthess J Pandey S Capitani M Rue-Albrecht KC Arnold I Franchini F. The Short Chain Fatty Acid Butyrate Imprints an Antimicrobial Program in Macrophages. Immunity (2019) 50(2):432–45.e7. 10.1016/j.immuni.2018.12.018 30683619 PMC6382411

[19] Martinez-Lopez M Iborra S Conde-Garrosa R Mastrangelo A Danne C Mann ER. Microbiota Sensing by Mincle-Syk Axis in Dendritic Cells Regulates Interleukin-17 and -22 Production and Promotes Intestinal Barrier Integrity. Immunity (2019) 50(2):446–61.e9. 10.1016/j.immuni.2018.12.020 30709742 PMC6382412

[20] Umesaki Y Setoyama H Matsumoto S Okada Y. Expansion of Alpha Beta T-cell Receptor-Bearing Intestinal Intraepithelial Lymphocytes After Microbial Colonization in Germ-Free Mice and its Independence From Thymus. Immunology (1993) 79(1):32–7. PMC1422052 8509140

[21] Ivanov II Frutos Rde L Manel N Yoshinaga K Rifkin DB Sartor RB. Specific Microbiota Direct the Differentiation of IL-17-producing T-Helper Cells in the Mucosa of the Small Intestine. Cell Host Microbe (2008) 4(4):337–49. 10.1016/j.chom.2008.09.009 PMC2597589 18854238

[22] Mazmanian SK Liu CH Tzianabos AO Kasper DL. An Immunomodulatory Molecule of Symbiotic Bacteria Directs Maturation of the Host Immune System. Cell (2005) 122(1):107–18. 10.1016/j.cell.2005.05.007 16009137

[23] Bachem A Makhlouf C Binger KJ de Souza DP Tull D Hochheiser K. Microbiota-Derived Short-Chain Fatty Acids Promote the Memory Potential of Antigen-Activated Cd8(+) T Cells. Immunity (2019) 51(2):285–97.e5. 10.1016/j.immuni.2019.06.002 31272808

[24] Song X Sun X Oh SF Wu M Zhang Y Zheng W. Microbial Bile Acid Metabolites Modulate Gut RORgamma(+) Regulatory T Cell Homeostasis. Nature (2020) 577(7790):410–5. 10.1038/s41586-019-1865-0 PMC7274525 31875848

[25] Iida N Dzutsev A Stewart CA Smith L Bouladoux N Weingarten RA. Commensal Bacteria Control Cancer Response to Therapy by Modulating the Tumor Microenvironment. Science (2013) 342(6161):967–70. 10.1126/science.1240527 PMC6709532 24264989

[26] Viaud S Saccheri F Mignot G Yamazaki T Daillere R Hannani D. The Intestinal Microbiota Modulates the Anticancer Immune Effects of Cyclophosphamide. Science (2013) 342(6161):971–6. 10.1126/science.1240537 PMC4048947 24264990

[27] Bradley SM Neumann VC Barr K Troughton PR Astbury C Bird HA. Sequential Study of Bacterial Antibody Levels and Faecal Flora in Rheumatoid Arthritis Patients Taking Sulphasalazine. Br J Rheumatol (1993) 32(8):683–8. 10.1093/rheumatology/32.8.683 8102304

[28] Ananthakrishnan AN Luo C Yajnik V Khalili H Garber JJ Stevens BW. Gut Microbiome Function Predicts Response to Anti-integrin Biologic Therapy in Inflammatory Bowel Diseases. Cell Host Microbe (2017) 21(5):603–10.e3. 10.1016/j.chom.2017.04.010 28494241 PMC5705050

[29] Zhou Y Xu ZZ He Y Yang Y Liu L Lin Q. Gut Microbiota Offers Universal Biomarkers Across Ethnicity in Inflammatory Bowel Disease Diagnosis and Infliximab Response Prediction. mSystems (2018) 3(1):e00188-17. 10.1128/mSystems.00188-17 29404425 PMC5790872

[30] Dewint P Hansen BE Verhey E Oldenburg B Hommes DW Pierik M. Adalimumab Combined With Ciprofloxacin is Superior to Adalimumab Monotherapy in Perianal Fistula Closure in Crohn’s Disease: A Randomised, Double-Blind, Placebo Controlled Trial (ADAFI). Gut (2014) 63(2):292–9. 10.1136/gutjnl-2013-304488 23525574

[31] Tang ML Ponsonby AL Orsini F Tey D Robinson M Su EL. Administration of a Probiotic With Peanut Oral Immunotherapy: A Randomized Trial. J Allergy Clin Immunol (2015) 135(3):737–44.e8. 10.1016/j.jaci.2014.11.034 25592987

[32] Wei SC Duffy CR Allison JP. Fundamental Mechanisms of Immune Checkpoint Blockade Therapy. Cancer Discovery (2018) 8(9):1069–86. 10.1158/2159-8290.CD-18-0367 30115704

[33] Darvin P Toor SM Sasidharan Nair V Elkord E. Immune Checkpoint Inhibitors: Recent Progress and Potential Biomarkers. Exp Mol Med (2018) 50(12):1–11. 10.1038/s12276-018-0191-1 PMC6292890 30546008

[34] Wilky BA. Immune Checkpoint Inhibitors: The Linchpins of Modern Immunotherapy. Immunol Rev (2019) 290(1):6–23. 10.1111/imr.12766 31355494

[35] Cai J Wang D Zhang G Guo X. The Role of PD-1/PD-L1 Axis In Treg Development and Function: Implications For Cancer Immunotherapy. Onco Targets Ther (2019) 12:8437–45. 10.2147/OTT.S221340 PMC6800566 31686860

[36] Chen DS Mellman I. Oncology Meets Immunology: The Cancer-Immunity Cycle. Elsevier (2013) p:1–10. 10.1016/j.immuni.2013.07.012 23890059

[37] Luu M Weigand K Wedi F Breidenbend C Leister H Pautz S. Regulation of the Effector Function of CD8(+) T Cells by Gut Microbiota-Derived Metabolite Butyrate. Sci Rep (2018) 8(1):14430. 10.1038/s41598-018-32860-x 30258117 PMC6158259

[38] Cremonesi E Governa V Garzon JFG Mele V Amicarella F Muraro MG. Gut Microbiota Modulate T Cell Trafficking Into Human Colorectal Cancer. Gut (2018) 67(11):1984–94. 10.1136/gutjnl-2016-313498 29437871

[39] Zitvogel L Daillere R Roberti MP Routy B Kroemer G. Anticancer Effects of the Microbiome and its Products. Nat Rev Microbiol (2017) 15(8):465–78. 10.1038/nrmicro.2017.44 28529325

[40] Smith PM Howitt MR Panikov N Michaud M Gallini CA Bohlooly YM. The Microbial Metabolites, Short-Chain Fatty Acids, Regulate Colonic Treg Cell Homeostasis. Science (2013) 341(6145):569–73. 10.1126/science.1241165 PMC3807819 23828891

[41] Hoeppli RE Wu D Cook L Levings MK. The Environment of Regulatory T Cell Biology: Cytokines, Metabolites, and the Microbiome. Front Immunol (2015) 6:61. 10.3389/fimmu.2015.00061 25741338 PMC4332351

[42] Poggi A Benelli R Venè R Costa D Ferrari N Tosetti F. Human Gut-Associated Natural Killer Cells in Health and Disease. Front Immunol (2019) 10:961. 10.3389/fimmu.2019.00961 31130953 PMC6509241

[43] Zitvogel L Ayyoub M Routy B Kroemer G. Microbiome and Anticancer Immunosurveillance. Cell (2016) 165(2):276–87. 10.1016/j.cell.2016.03.001 27058662

[44] Andre FE Booy R Bock HL Clemens J Datta SK John TJ. Vaccination Greatly Reduces Disease, Disability, Death and Inequity Worldwide. Bull World Health Organ (2008) 86(2):140–6. 10.2471/BLT.07.040089 PMC2647387 18297169

[45] Pezzotti P Bellino S Prestinaci F Iacchini S Lucaroni F Camoni L. The Impact of Immunization Programs on 10 Vaccine Preventable Diseases in Italy: 1900-2015. Vaccine (2018) 36(11):1435–43. 10.1016/j.vaccine.2018.01.065 29428176

[46] Sallusto F Lanzavecchia A Araki K Ahmed R. From Vaccines to Memory and Back. Immunity (2010) 33(4):451–63. 10.1016/j.immuni.2010.10.008 PMC3760154 21029957

[47] McNeela EA Mills KH. Manipulating the Immune System: Humoral Versus Cell-Mediated Immunity. Adv Drug Delivery Rev (2001) 51(1-3):43–54. 10.1016/S0169-409X(01)00169-7 11516778

[48] Kleindienst P Brocker T. Endogenous Dendritic Cells are Required for Amplification of T Cell Responses Induced by Dendritic Cell Vaccines In Vivo. J Immunol (2003) 170(6):2817–23. 10.4049/jimmunol.170.6.2817 12626531

[49] Serre K Cunningham AF Coughlan RE Lino AC Rot A Hub E. Cd8 T Cells Induce T-bet-dependent Migration Toward CXCR3 Ligands by Differentiated B Cells Produced During Responses to Alum-Protein Vaccines. Blood (2012) 120(23):4552–9. 10.1182/blood-2012-03-417733 23065152

[50] Steinman RM. Dendritic Cells In Vivo: A Key Target for a New Vaccine Science. Immunity (2008) 29(3):319–24. 10.1016/j.immuni.2008.08.001 18799140

[51] Havenar-Daughton C Abbott RK Schief WR Crotty S. When Designing Vaccines, Consider the Starting Material: The Human B Cell Repertoire. Curr Opin Immunol (2018) 53:209–16. 10.1016/j.coi.2018.08.002 PMC6148213 30190230

[52] Ubeda C Pamer EG. Antibiotics, Microbiota, and Immune Defense. Trends Immunol (2012) 33(9):459–66. 10.1016/j.it.2012.05.003 PMC3427468 22677185

[53] Pamer EG. Microbial Tuning of the Mammalian Immune System. Trends Mol Med (2017) 23(5):379–80. 10.1016/j.molmed.2017.03.006 PMC5428386 28372921

[54] Schirmer M Smeekens SP Vlamakis H Jaeger M Oosting M Franzosa EA. Linking the Human Gut Microbiome to Inflammatory Cytokine Production Capacity. Cell (2016) 167(7):1897. 10.1016/j.cell.2016.11.046 27984736

[55] Li B Chan HL Chen P. Immune Checkpoint Inhibitors: Basics and Challenges. Curr Med Chem (2019) 26(17):3009–25. 10.2174/0929867324666170804143706 28782469

[56] Havel JJ Chowell D Chan TA. The Evolving Landscape of Biomarkers for Checkpoint Inhibitor Immunotherapy. Nat Rev Cancer (2019) 19(3):133–50. 10.1038/s41568-019-0116-x PMC6705396 30755690

[57] Gibney GT Weiner LM Atkins MB. Predictive Biomarkers for Checkpoint Inhibitor-Based Immunotherapy. Lancet Oncol (2016) 17(12):e542–e51. 10.1016/S1470-2045(16)30406-5 PMC5702534 27924752

[58] Routy B Le Chatelier E Derosa L Duong CPM Alou MT Daillère R. Gut Microbiome Influences Efficacy of PD-1–based Immunotherapy Against Epithelial Tumors. Science (2018) 359(6371):91–7. 10.1126/science.aan3706 29097494

[59] Chaput N Lepage P Coutzac C Soularue E Le Roux K Monot C. Baseline Gut Microbiota Predicts Clinical Response and Colitis in Metastatic Melanoma Patients Treated With Ipilimumab. Ann Oncol (2017) 28(6):1368–79. 10.1093/annonc/mdx108 28368458

[60] Frankel AE Coughlin LA Kim J Froehlich TW Xie Y Frenkel EP. Metagenomic Shotgun Sequencing and Unbiased Metabolomic Profiling Identify Specific Human Gut Microbiota and Metabolites Associated With Immune Checkpoint Therapy Efficacy in Melanoma Patients 1. Neoplasia (2017) 19:848–55. 10.1016/j.neo.2017.08.004 PMC5602478 28923537

[61] Gopalakrishnan V Spencer CN Nezi L Reuben A Andrews MC Karpinets TV. Gut Microbiome Modulates Response to Anti–PD-1 Immunotherapy in Melanoma Patients. Science (2018) 359(6371):97–103. 10.1126/science.aan4236 29097493 PMC5827966

[62] Matson V Fessler J Bao R Chongsuwat T Zha Y Alegre M-L. The Commensal Microbiome is Associated With Anti–PD-1 Efficacy in Metastatic Melanoma Patients. Science (2018) 359(6371):104–8. 10.1126/science.aao3290 PMC6707353 29302014

[63] Coutzac C Jouniaux J-M Paci A Schmidt J Mallardo D Seck A. Systemic Short Chain Fatty Acids Limit Antitumor Effect of CTLA-4 Blockade in Hosts With Cancer. Nat Commun (2020) 11(1):2168–. 10.1038/s41467-020-16079-x PMC7195489 32358520

[64] Peters BA Wilson M Moran U Pavlick A Izsak A Wechter T. Relating the Gut Metagenome and Metatranscriptome to Immunotherapy Responses in Melanoma Patients. Genome Med (2019) 11(1). 10.1186/s13073-019-0672-4 PMC6785875 31597568

[65] Derosa L Hellmann MD Spaziano M Halpenny D Fidelle M Rizvi H. Negative Association of Antibiotics on Clinical Activity of Immune Checkpoint Inhibitors in Patients With Advanced Renal Cell and non-Small-Cell Lung Cancer. Ann Oncol (2018) 29(6):1437–44. 10.1093/annonc/mdy103 PMC6354674 29617710

[66] Derosa L Routy B Fidelle M Iebba V Alla L Pasolli E. Gut Bacteria Composition Drives Primary Resistance to Cancer Immunotherapy in Renal Cell Carcinoma Patients. Eur Urol (2020) 78(2):195–206. 10.1016/j.eururo.2020.04.044 32376136

[67] Zhao S Gao G Li W Li X Zhao C Jiang T. Antibiotics are Associated With Attenuated Efficacy of anti-PD-1/PD-L1 Therapies in Chinese Patients With Advanced non-Small Cell Lung Cancer. Lung Cancer (2019) 130:10–7. 10.1016/j.lungcan.2019.01.017 30885328

[68] Mohiuddin JJ Chu B Facciabene A Poirier K Wang X Doucette A. Association of Antibiotic Exposure With Survival and Toxicity in Patients With Melanoma Receiving Immunotherapy. J Natl Cancer Inst (2020) 113(2):162–70. 10.1093/jnci/djaa057 PMC7850522 32294209

[69] Lurienne L Cervesi J Duhalde L de Gunzburg J Andremont A Zalcman G. Non-Small-Cell Lung Cancer Immunotherapy Efficacy and Antibiotic Use: A Systematic Review and Meta-Analysis. J Thorac Oncol (2020) 15(7):1147–59. 10.1016/j.jtho.2020.03.002 32173463

[70] Kaderbhai C Richard C Fumet JD Aarnink A Foucher P Coudert B. Antibiotic Use Does Not Appear to Influence Response to Nivolumab. Anticancer Res (2017) 37(6):3195–200. 10.21873/anticanres.11680 28551664

[71] Tomita Y Ikeda T Sakata S Saruwatari K Sato R Iyama S. Association of Probiotic Clostridium Butyricum Therapy With Survival and Response to Immune Checkpoint Blockade in Patients With Lung Cancer. Cancer Immunol Res (2020) 8(10):1236–42. 10.1158/2326-6066.CIR-20-0051 32665261

[72] Sokol H Pigneur B Watterlot L Lakhdari O Bermudez-Humaran LG Gratadoux JJ. Faecalibacterium Prausnitzii is an Anti-Inflammatory Commensal Bacterium Identified by Gut Microbiota Analysis of Crohn Disease Patients. Proc Natl Acad Sci USA (2008) 105(43):16731–6. 10.1073/pnas.0804812105 PMC2575488 18936492

[73] Galecka M Szachta P Bartnicka A Lykowska-Szuber L Eder P Schwiertz A. Faecalibacterium Prausnitzii and Crohn’s Disease - is There Any Connection? Pol J Microbiol (2013) 62(1):91–5. 10.33073/pjm-2013-013 23829084

[74] Song H Yoo Y Hwang J Na YC Kim HS. Faecalibacterium Prausnitzii Subspecies-Level Dysbiosis in the Human Gut Microbiome Underlying Atopic Dermatitis. J Allergy Clin Immunol (2016) 137(3):852–60. 10.1016/j.jaci.2015.08.021 26431583

[75] Geva-Zatorsky N Sefik E Kua L Pasman L Tan TG Ortiz-Lopez A. Mining the Human Gut Microbiota for Immunomodulatory Organisms. Cell (2017) 168(5):928–43. 10.1016/j.cell.2017.01.022 PMC7774263 28215708

[76] Fluckiger A Daillere R Sassi M Sixt BS Liu P Loos F. Cross-Reactivity Between Tumor MHC Class I-restricted Antigens and an Enterococcal Bacteriophage. Science (2020) 369(6506):936–42. 10.1126/science.aax0701 32820119

[77] Shen H Yang ESH Conry M Fiveash J Contreras C Bonner JA. Predictive Biomarkers for Immune Checkpoint Blockade and Opportunities for Combination Therapies. Genes & Diseases (2019) 6(3):232–46. 10.1016/j.gendis.2019.06.006 PMC6997608 32042863

[78] Lérias JR Paraschoudi G de Sousa E Martins J Condeço C Figueiredo N. Microbes as Master Immunomodulators: Immunopathology, Cancer and Personalized Immunotherapies. Front Cell Dev Biol (2020) 7:362–. 10.3389/fcell.2019.00362 PMC6989410 32039196

[79] Poore GD Kopylova E Zhu Q Carpenter C Fraraccio S Wandro S. Microbiome Analyses of Blood and Tissues Suggest Cancer Diagnostic Approach. Nature (2020) 579(7800):567–74. 10.1038/s41586-020-2095-1 PMC7500457 32214244

[80] Gavrielatou N Doumas S Economopoulou P Foukas PG Psyrri A. Biomarkers for Immunotherapy Response in Head and Neck Cancer. Cancer Treat Rev (2020) 84:101977. 10.1016/j.ctrv.2020.101977 32018128

[81] Adlung L Elinav E Greten TF Korangy F. Microbiome Genomics for Cancer Prediction. Nat Cancer (2020) 1(4):379–81. 10.1038/s43018-020-0059-x 35121970

[82] Sivan A Corrales L Hubert N Williams JB Aquino-Michaels K Earley ZM. Commensal Bifidobacterium Promotes Antitumor Immunity and Facilitates anti-PD-L1 Efficacy. Science (2015) 350(6264):1084–9. 10.1126/science.aac4255 PMC4873287 26541606

[83] Vétizou M Pitt JM Daillère R Lepage P Waldschmitt N Flament C. Anticancer Immunotherapy by CTLA-4 Blockade Relies on the Gut Microbiota. Science (2015) 350(6264):1079–84. 10.1126/science.aad1329 PMC4721659 26541610

[84] Tanoue T Morita S Plichta D Skelly AN Suda W Sugiura Y. A Defined Commensal Consortium Elicits CD8 T Cells and Anti-Cancer Immunity. Nature (2019) 565(7741):600–5. 10.1038/s41586-019-0878-z 30675064

[85] Xu X Lv J Guo F Li J Jia Y Jiang D. Gut Microbiome Influences the Efficacy of PD-1 Antibody Immunotherapy on MSS-Type Colorectal Cancer Via Metabolic Pathway. Front Microbiol (2020) 11:814. 10.3389/fmicb.2020.00814 32425919 PMC7212380

[86] Mager LF Burkhard R Pett N Cooke NCA Brown K Ramay H. Microbiome-Derived Inosine Modulates Response to Checkpoint Inhibitor Immunotherapy. Science (2020) 369(6510):eabc3421. 10.1126/science.abc3421 32792462

[87] Graber CD Goust JM Glassman AD Kendall R Loadholt CB. Immunomodulating Properties of Dimethylglycine in Humans. J Infect Dis (1981) 143(1):101–5. 10.1093/infdis/143.1.101 6163829

[88] Thurnher M Gruenbacher G. T Lymphocyte Regulation by Mevalonate Metabolism. Sci Signal (2015) 8(370):re4. 10.1126/scisignal.2005970 25829448

[89] Jin Y Dong H Xia L Yang Y Zhu Y Shen Y. The Diversity of Gut Microbiome is Associated With Favorable Responses to Anti–Programmed Death 1 Immunotherapy in Chinese Patients With Nsclc. J Thorac Oncol (2019) 14(8):1378–89. 10.1016/j.jtho.2019.04.007 31026576

[90] Gur C Ibrahim Y Isaacson B Yamin R Abed J Gamliel M. Binding of the Fap2 Protein of Fusobacterium Nucleatum to Human Inhibitory Receptor Tigit Protects Tumors From Immune Cell Attack. Immunity (2015) 42(2):344–55. 10.1016/j.immuni.2015.01.010 PMC4361732 25680274

[91] Gur C Maalouf N Shhadeh A Berhani O Singer BB Bachrach G. Fusobacterium Nucleatum Supresses Anti-Tumor Immunity by Activating CEACAM1. OncoImmunology (2019) 8(6):e1581531. 10.1080/2162402X.2019.1581531 31069151 PMC6492956

[92] Kasper SH Morell-Perez C Wyche TP Sana TR Lieberman LA Hett EC. Colorectal Cancer-Associated Anaerobic Bacteria Proliferate in Tumor Spheroids and Alter the Microenvironment. Sci Rep (2020) 10(1):1–13. 10.1038/s41598-020-62139-z 32210258 PMC7093526

[93] Nejman D Livyatan I Fuks G Gavert N Zwang Y Geller LT. The Human Tumor Microbiome is Composed of Tumor Type–Specific Intracellular Bacteria. Science (2020) 368(6494):973–80. 10.1126/science.aay9189 PMC7757858 32467386

[94] Busquets D Mas-de-Xaxars T Lopez-Siles M Martinez-Medina M Bahi A Sabat M. Anti-Tumour Necrosis Factor Treatment With Adalimumab Induces Changes in the Microbiota of Crohn’s Disease. J Crohns Colitis (2015) 9(10):899–906. 10.1093/ecco-jcc/jjv119 26142465

[95] Wang Y Gao X Ghozlane A Hu H Li X Xiao Y. Characteristics of Faecal Microbiota in Paediatric Crohn’s Disease and Their Dynamic Changes During Infliximab Therapy. J Crohns Colitis (2018) 12(3):337–46. 10.1093/ecco-jcc/jjx153 29194468

[96] Angelakis E Million M Kankoe S Lagier JC Armougom F Giorgi R. Abnormal Weight Gain and Gut Microbiota Modifications are Side Effects of Long-Term Doxycycline and Hydroxychloroquine Treatment. Antimicrob Agents Chemother (2014) 58(6):3342–7. 10.1128/AAC.02437-14 PMC4068504 24687497

[97] Zaza G Dalla Gassa A Felis G Granata S Torriani S Lupo A. Impact of Maintenance Immunosuppressive Therapy on the Fecal Microbiome of Renal Transplant Recipients: Comparison Between an Everolimus- and a Standard Tacrolimus-Based Regimen. PloS One (2017) 12(5):e0178228. 10.1371/journal.pone.0178228 28542523 PMC5443527

[98] Ostrov BE Amsterdam D. Immunomodulatory Interplay of the Microbiome and Therapy of Rheumatic Diseases. Immunol Invest (2017) 46(8):769–92. 10.1080/08820139.2017.1373828 29058546

[99] Som A Mandaliya R Alsaadi D Farshidpour M Charabaty A Malhotra N. Immune Checkpoint Inhibitor-Induced Colitis: A Comprehensive Review. World J Clin cases (2019) 7(4):405–18. 10.12998/wjcc.v7.i4.405 PMC6397821 30842952

[100] Baruch EN Youngster I Ben-Betzalel G Ortenberg R Lahat A Katz L. Fecal Microbiota Transplant Promotes Response in Immunotherapy-Refractory Melanoma Patients. Science (2020) 371(6529):602–9. 10.1126/science.abb5920 33303685

[101] Wang Y Wiesnoski DH Helmink BA Gopalakrishnan V Choi K DuPont HL. Fecal Microbiota Transplantation for Refractory Immune Checkpoint Inhibitor-Associated Colitis. Nat Med (2018) 24(12):1804–8. 10.1038/s41591-018-0238-9 PMC6322556 30420754

[102] Marinelli L Tenore GC Novellino E. Probiotic Species in the Modulation of the Anticancer Immune Response. Semin Cancer Biol (2017) 46:182–90. 10.1016/j.semcancer.2017.08.007 28844794

[103] Pulendran B. Systems Vaccinology: Probing Humanity’s Diverse Immune Systems With Vaccines. Proc Natl Acad Sci USA (2014) 111(34):12300–6. 10.1073/pnas.1400476111 PMC4151766 25136102

[104] Wiedermann U Garner-Spitzer E Wagner A. Primary Vaccine Failure to Routine Vaccines: Why and What to do? Hum Vaccin Immunother (2016) 12(1):239–43. 10.1080/21645515.2015.1093263 PMC4962729 26836329

[105] McDermott AB Cohen SB Zuckerman JN Madrigal JA. Hepatitis B Third-Generation Vaccines: Improved Response and Conventional Vaccine non-Response–Evidence for Genetic Basis in Humans. J Viral Hepat (1998) 5 Suppl 2:9–11. 10.1046/j.1365-2893.1998.0050s2009.x 9857354

[106] Gelder CM Lambkin R Hart KW Fleming D Williams OM Bunce M. Associations Between Human Leukocyte Antigens and Nonresponsiveness to Influenza Vaccine. J Infect Dis (2002) 185(1):114–7. 10.1086/338014 11756990

[107] Fisman DN Agrawal D Leder K. The Effect of Age on Immunologic Response to Recombinant Hepatitis B Vaccine: A Meta-Analysis. Clin Infect Dis (2002) 35(11):1368–75. 10.1086/344271 12439800

[108] Chen WH Kozlovsky BF Effros RB Grubeck-Loebenstein B Edelman R Sztein MB. Vaccination in the Elderly: An Immunological Perspective. Trends Immunol (2009) 30(7):351–9. 10.1016/j.it.2009.05.002 PMC3739436 19540808

[109] McElhaney JE Zhou X Talbot HK Soethout E Bleackley RC Granville DJ. The Unmet Need in the Elderly: How Immunosenescence, CMV Infection, Co-Morbidities and Frailty are a Challenge for the Development of More Effective Influenza Vaccines. Vaccine (2012) 30(12):2060–7. 10.1016/j.vaccine.2012.01.015 PMC3345132 22289511

[110] Yang S Tian G Cui Y Ding C Deng M Yu C. Factors Influencing Immunologic Response to Hepatitis B Vaccine in Adults. Sci Rep (2016) 6:27251. 10.1038/srep27251 27324884 PMC4914839

[111] Trzonkowski P Mysliwska J Szmit E Wieckiewicz J Lukaszuk K Brydak LB. Association Between Cytomegalovirus Infection, Enhanced Proinflammatory Response and Low Level of Anti-Hemagglutinins During the Anti-Influenza Vaccination–an Impact of Immunosenescence. Vaccine (2003) 21(25-26):3826–36. 10.1016/S0264-410X(03)00309-8 12922116

[112] Pasricha N Datta U Chawla Y Singh S Arora SK Sud A. Immune Responses in Patients With HIV Infection After Vaccination With Recombinant Hepatitis B Virus Vaccine. BMC Infect Dis (2006) 6:65. 10.1186/1471-2334-6-65 16571140 PMC1525180

[113] Akmatov MK Riese P Trittel S May M Prokein J Illig T. Self-Reported Diabetes and Herpes Zoster are Associated With a Weak Humoral Response to the Seasonal Influenza A H1N1 Vaccine Antigen Among the Elderly. BMC Infect Dis (2019) 19(1):656. 10.1186/s12879-019-4214-x 31337344 PMC6651912

[114] Sheridan PA Paich HA Handy J Karlsson EA Hudgens MG Sammon AB. Obesity is Associated With Impaired Immune Response to Influenza Vaccination in Humans. Int J Obes (Lond) (2012) 36(8):1072–7. 10.1038/ijo.2011.208 PMC3270113 22024641

[115] Haque R Snider C Liu Y Ma JZ Liu L Nayak U. Oral Polio Vaccine Response in Breast Fed Infants With Malnutrition and Diarrhea. Vaccine (2014) 32(4):478–82. 10.1016/j.vaccine.2013.11.056 PMC4936916 24300591

[116] Eleftheriadis T Antoniadi G Liakopoulos V Kartsios C Stefanidis I. Disturbances of Acquired Immunity in Hemodialysis Patients. Semin Dial (2007) 20(5):440–51. 10.1111/j.1525-139X.2007.00283.x 17897251

[117] Noh KW Poland GA Murray JA. Hepatitis B Vaccine Nonresponse and Celiac Disease. Am J Gastroenterol (2003) 98(10):2289–92. 10.1111/j.1572-0241.2003.07701.x 14572581

[118] Patel DP Treat JR Castelo-Socio L. Decreased Hepatitis B Vaccine Response in Pediatric Patients With Atopic Dermatitis, Psoriasis, and Morphea. Vaccine (2017) 35(35 Pt B):4499–500. 10.1016/j.vaccine.2017.07.025 28736199

[119] Sell S. Mercaptoethanol-Sensitive Antibody Production in Germ-Free Mice and Guinea Pigs. J Immunol (1965) 95(2):300–5. 4158331

[120] Porter P Kenworthy R Noakes DE Allen WD. Intestinal Antibody Secretion in the Young Pig in Response to Oral Immunization With Escherichia Coli. Immunology (1974) 27(5):841–53. PMC1445675 4611908

[121] Ohwaki M Yasutake N Yasui H Ogura R. A Comparative Study on the Humoral Immune Responses in Germ-Free and Conventional Mice. Immunology (1977) 32(1):43–8. PMC1445201 321340

[122] Parry SH Allen WD Porter P. Intestinal Immune Response to E. coli antigens in the germ-free chicken. Immunology (1977) 32(5):731–41. PMC1445302 324901

[123] MacDonald TT Carter PB. Requirement for a Bacterial Flora Before Mice Generate Cells Capable of Mediating the Delayed Hypersensitivity Reaction to Sheep Red Blood Cells. J Immunol (1979) 122(6):2624–9. 448137

[124] Hobby GL Lenert TF Maier-Engallena J Wakely-Oliver C Manty A Cicenia E. Further Observations on the Immunogenic Effect of BCG in Germfree Mice. II. Am Rev Respir Dis (1968) 97(6):1095–103. 10.1164/arrd.1968.97.6P1.1095 4870218

[125] Lamouse-Smith ES Tzeng A Starnbach MN. The Intestinal Flora is Required to Support Antibody Responses to Systemic Immunization in Infant and Germ Free Mice. PloS One (2011) 6(11):e27662. 10.1371/journal.pone.0027662 22114681 PMC3219679

[126] Oh JZ Ravindran R Chassaing B Carvalho FA Maddur MS Bower M. TLR5-Mediated Sensing of Gut Microbiota is Necessary for Antibody Responses to Seasonal Influenza Vaccination. Immunity (2014) 41(3):478–92. 10.1016/j.immuni.2014.08.009 PMC4169736 25220212

[127] Uchiyama R Chassaing B Zhang B Gewirtz AT. Antibiotic Treatment Suppresses Rotavirus Infection and Enhances Specific Humoral Immunity. J Infect Dis (2014) 210(2):171–82. 10.1093/infdis/jiu037 PMC4399425 24436449

[128] Kim D Kim YG Seo SU Kim DJ Kamada N Prescott D. Nod2-mediated Recognition of the Microbiota is Critical for Mucosal Adjuvant Activity of Cholera Toxin. Nat Med (2016) 22(5):524–30. 10.1038/nm.4075 PMC4860092 27064448

[129] Kim D Kim YM Kim WU Park JH Nunez G Seo SU. Recognition of the Microbiota by Nod2 Contributes to the Oral Adjuvant Activity of Cholera Toxin Through the Induction of Interleukin-1beta. Immunology (2019) 158(3):219–29. 10.1111/imm.13105 PMC6797898 31478196

[130] Woo PC Tsoi HW Wong LP Leung HC Yuen KY. Antibiotics Modulate Vaccine-Induced Humoral Immune Response. Clin Diagn Lab Immunol (1999) 6(6):832–7. 10.1128/CDLI.6.6.832-837.1999 PMC95784 10548572

[131] Lynn MA Tumes DJ Choo JM Sribnaia A Blake SJ Leong LEX. Early-Life Antibiotic-Driven Dysbiosis Leads to Dysregulated Vaccine Immune Responses in Mice. Cell Host Microbe (2018) 23(5):653–60.e5. 10.1016/j.chom.2018.04.009 29746836

[132] Yang W Xiao Y Huang X Chen F Sun M Bilotta AJ. Microbiota Metabolite Short-Chain Fatty Acids Facilitate Mucosal Adjuvant Activity of Cholera Toxin Through GPR43. J Immunol (2019) 203(1):282–92. 10.4049/jimmunol.1801068 PMC6581581 31076530

[133] Zhang Y Wu Q Zhou M Luo Z Lv L Pei J. Composition of the Murine Gut Microbiome Impacts Humoral Immunity Induced by Rabies Vaccines. Clin Transl Med (2020) 10(4):e161. 10.1002/ctm2.161 32898335 PMC7443138

[134] Vos AP Haarman M Buco A Govers M Knol J Garssen J. A Specific Prebiotic Oligosaccharide Mixture Stimulates Delayed-Type Hypersensitivity in a Murine Influenza Vaccination Model. Int Immunopharmacol (2006) 6(8):1277–86. 10.1016/j.intimp.2006.03.010 16782540

[135] Benyacoub J Rochat F Saudan KY Rochat I Antille N Cherbut C. Feeding a Diet Containing a Fructooligosaccharide Mix can Enhance Salmonella Vaccine Efficacy in Mice. J Nutr (2008) 138(1):123–9. 10.1093/jn/138.1.123 18156414

[136] Vos AP Knol J Stahl B M’Rabet L Garssen J. Specific Prebiotic Oligosaccharides Modulate the Early Phase of a Murine Vaccination Response. Int Immunopharmacol (2010) 10(5):619–25. 10.1016/j.intimp.2010.02.014 20206301

[137] van den Elsen LWJ Tims S Jones AM Stewart A Stahl B Garssen J. Prebiotic Oligosaccharides in Early Life Alter Gut Microbiome Development in Male Mice While Supporting Influenza Vaccination Responses. Benef Microbes (2019) 10(3):279–91. 10.3920/BM2018.0098 30773928

[138] Di Luccia B Ahern PP Griffin NW Cheng J Guruge JL Byrne AE. Combined Prebiotic and Microbial Intervention Improves Oral Cholera Vaccination Responses in a Mouse Model of Childhood Undernutrition. Cell Host Microbe (2020) 27(6):899–908 e5. 10.1016/j.chom.2020.04.008 32348782 PMC7292785

[139] Wen K Li G Bui T Liu F Li Y Kocher J. High Dose and Low Dose Lactobacillus Acidophilus Exerted Differential Immune Modulating Effects on T Cell Immune Responses Induced by an Oral Human Rotavirus Vaccine in Gnotobiotic Pigs. Vaccine (2012) 30(6):1198–207. 10.1016/j.vaccine.2011.11.107 PMC3269528 22178726

[140] Chattha KS Vlasova AN Kandasamy S Rajashekara G Saif LJ. Divergent Immunomodulating Effects of Probiotics on T Cell Responses to Oral Attenuated Human Rotavirus Vaccine and Virulent Human Rotavirus Infection in a Neonatal Gnotobiotic Piglet Disease Model. J Immunol (2013) 191(5):2446–56. 10.4049/jimmunol.1300678 PMC4136549 23918983

[141] Kandasamy S Chattha KS Vlasova AN Rajashekara G Saif LJ. Lactobacilli and Bifidobacteria Enhance Mucosal B Cell Responses and Differentially Modulate Systemic Antibody Responses to an Oral Human Rotavirus Vaccine in a Neonatal Gnotobiotic Pig Disease Model. Gut Microbes (2014) 5(5):639–51. 10.4161/19490976.2014.969972 PMC4615723 25483333

[142] Wen K Tin C Wang H Yang X Li G Giri-Rachman E. Probiotic Lactobacillus Rhamnosus GG Enhanced Th1 Cellular Immunity But did Not Affect Antibody Responses in a Human Gut Microbiota Transplanted Neonatal Gnotobiotic Pig Model. PloS One (2014) 9(4):e94504. 10.1371/journal.pone.0094504 24722168 PMC3983166

[143] Arnold IC Hutchings C Kondova I Hey A Powrie F Beverley P. Helicobacter Hepaticus Infection in BALB/c Mice Abolishes Subunit-Vaccine-Induced Protection Against M. Tuberculosis. Vaccine (2015) 33(15):1808–14. 10.1016/j.vaccine.2015.02.041 PMC4377097 25748336

[144] Reese TA Bi K Kambal A Filali-Mouhim A Beura LK Burger MC. Sequential Infection With Common Pathogens Promotes Human-Like Immune Gene Expression and Altered Vaccine Response. Cell Host Microbe (2016) 19(5):713–9. 10.1016/j.chom.2016.04.003 PMC4896745 27107939

[145] Stebegg M Silva-Cayetano A Innocentin S Jenkins TP Cantacessi C Gilbert C. Heterochronic Faecal Transplantation Boosts Gut Germinal Centres in Aged Mice. Nat Commun (2019) 10(1):2443. 10.1038/s41467-019-10430-7 31164642 PMC6547660

[146] Bumgardner SA Zhang L LaVoy AS Andre B Frank CB Kajikawa A. Nod2 is Required for Antigen-Specific Humoral Responses Against Antigens Orally Delivered Using a Recombinant Lactobacillus Vaccine Platform. PloS One (2018) 13(5):e0196950. 10.1371/journal.pone.0196950 29734365 PMC5937747

[147] Parker EP Ramani S Lopman BA Church JA Iturriza-Gomara M Prendergast AJ. Causes of Impaired Oral Vaccine Efficacy in Developing Countries. Future Microbiol (2018) 13:97–118. 10.2217/fmb-2017-0128 29218997 PMC7026772

[148] Jamieson AM. Influence of the Microbiome on Response to Vaccination. Hum Vaccin Immunother (2015) 11(9):2329–31. 10.1080/21645515.2015.1022699 PMC4635895 26090701

[149] Valdez Y Brown EM Finlay BB. Influence of the Microbiota on Vaccine Effectiveness. Trends Immunol (2014) 35(11):526–37. 10.1016/j.it.2014.07.003 25113637

[150] Blacher E Levy M Tatirovsky E Elinav E. Microbiome-Modulated Metabolites At the Interface of Host Immunity. J Immunol (2017) 198(2):572–80. 10.4049/jimmunol.1601247 28069752

[151] Lynn DJ Pulendran B. The Potential of the Microbiota to Influence Vaccine Responses. J Leukoc Biol (2018) 103(2):225–31. 10.1189/jlb.5MR0617-216R PMC5921907 28864446

[152] Ciabattini A Olivieri R Lazzeri E Medaglini D. Role of the Microbiota in the Modulation of Vaccine Immune Responses. Front Microbiol (2019) 10:1305. 10.3389/fmicb.2019.01305 31333592 PMC6616116

[153] de Jong SE Olin A Pulendran B. The Impact of the Microbiome on Immunity to Vaccination in Humans. Cell Host Microbe (2020) 28(2):169–79. 10.1016/j.chom.2020.06.014 PMC7422826 32791110

[154] Huda MN Lewis Z Kalanetra KM Rashid M Ahmad SM Raqib R. Stool Microbiota and Vaccine Responses of Infants. Pediatrics (2014) 134(2):e362–72. 10.1542/peds.2013-3937 PMC4187229 25002669

[155] Levine MM. Immunogenicity and Efficacy of Oral Vaccines in Developing Countries: Lessons From a Live Cholera Vaccine. BMC Biol (2010) 8:129. 10.1186/1741-7007-8-129 20920375 PMC2958895

[156] Becker-Dreps S Vilchez S Bucardo F Twitchell E Choi WS Hudgens MG. The Association Between Fecal Biomarkers of Environmental Enteropathy and Rotavirus Vaccine Response in Nicaraguan Infants. Pediatr Infect Dis J (2017) 36(4):412–6. 10.1097/INF.0000000000001457 27977553

[157] Praharaj I Parker EPK Giri S Allen DJ Silas S Revathi R. Influence of Nonpolio Enteroviruses and the Bacterial Gut Microbiota on Oral Poliovirus Vaccine Response: A Study From South India. J Infect Dis (2019) 219(8):1178–86. 10.1093/infdis/jiy568 PMC6601701 30247561

[158] Zhao T Li J Fu Y Ye H Liu X Li G. Influence of Gut Microbiota on Mucosal IgA Antibody Response to the Polio Vaccine. NPJ Vaccines (2020) 5:47. 10.1038/s41541-020-0194-5 PMC7283253 32566258

[159] Huda MN Ahmad SM Alam MJ Khanam A Kalanetra KM Taft DH. Bifidobacterium Abundance in Early Infancy and Vaccine Response At 2 Years of Age. Pediatrics (2019) 143(2):e20181489. 10.1542/peds.2018-1489 30674610 PMC6361348

[160] Twitchell EL Tin C Wen K Zhang H Becker-Dreps S Azcarate-Peril MA. Modeling Human Enteric Dysbiosis and Rotavirus Immunity in Gnotobiotic Pigs. Gut Pathog (2016) 8:51. 10.1186/s13099-016-0136-y 27826359 PMC5100090

[161] Goenka A Kollmann TR. Development of Immunity in Early Life. J Infect (2015) 71 Suppl 1:S112–20. 10.1016/j.jinf.2015.04.027 25934325

[162] Grassly NC Praharaj I Babji S Kaliappan SP Giri S Venugopal S. The Effect of Azithromycin on the Immunogenicity of Oral Poliovirus Vaccine: A Double-Blind Randomised Placebo-Controlled Trial in Seronegative Indian Infants. Lancet Infect Dis (2016) 16(8):905–14. 10.1016/S1473-3099(16)30023-8 27156189

[163] Harris VC Armah G Fuentes S Korpela KE Parashar U Victor JC. Significant Correlation Between the Infant Gut Microbiome and Rotavirus Vaccine Response in Rural Ghana. J Infect Dis (2017) 215(1):34–41. 10.1093/infdis/jiw518 27803175 PMC5225256

[164] Harris V Ali A Fuentes S Korpela K Kazi M Tate J. Rotavirus Vaccine Response Correlates With the Infant Gut Microbiota Composition in Pakistan. Gut Microbes (2018) 9(2):93–101. 10.1080/19490976.2017.1376162 28891751 PMC5989807

[165] Parker EPK Praharaj I Zekavati A Lazarus RP Giri S Operario DJ. Influence of the Intestinal Microbiota on the Immunogenicity of Oral Rotavirus Vaccine Given to Infants in South India. Vaccine (2018) 36(2):264–72. 10.1016/j.vaccine.2017.11.031 PMC5755003 29217369

[166] Fix J Chandrashekhar K Perez J Bucardo F Hudgens MG Yuan L. Association Between Gut Microbiome Composition and Rotavirus Vaccine Response Among Nicaraguan Infants. Am J Trop Med Hyg (2020) 102(1):213–9. 10.4269/ajtmh.19-0355 PMC6947780 31802728

[167] Harris VC Haak BW Handley SA Jiang B Velasquez DE Hykes BL Jr. Effect of Antibiotic-Mediated Microbiome Modulation on Rotavirus Vaccine Immunogenicity: A Human, Randomized-Control Proof-of-Concept Trial. Cell Host Microbe (2018) 24(2):197–207.e4. 10.1016/j.chom.2018.07.005 30092197 PMC11514417

[168] Eloe-Fadrosh EA McArthur MA Seekatz AM Drabek EF Rasko DA Sztein MB. Impact of Oral Typhoid Vaccination on the Human Gut Microbiota and Correlations With s. Typhi-specific immunological responses. PloS One (2013) 8(4):e62026. 10.1371/journal.pone.0062026 23637957 PMC3634757

[169] Salk HM Simon WL Lambert ND Kennedy RB Grill DE Kabat BF. Taxa of the Nasal Microbiome Are Associated With Influenza-Specific Iga Response to Live Attenuated Influenza Vaccine. PloS One (2016) 11(9):e0162803. 10.1371/journal.pone.0162803 27643883 PMC5028048

[170] Zimmermann P Curtis N. The Influence of the Intestinal Microbiome on Vaccine Responses. Vaccine (2018) 36(30):4433–9. 10.1016/j.vaccine.2018.04.066 29909134

[171] Hagan T Cortese M Rouphael N Boudreau C Linde C Maddur MS. Antibiotics-Driven Gut Microbiome Perturbation Alters Immunity to Vaccines in Humans. Cell (2019) 178(6):1313–28.e13. 10.1016/j.cell.2019.08.010 31491384 PMC6750738

[172] Zmora N Zilberman-Schapira G Suez J Mor U Dori-Bachash M Bashiardes S. Personalized Gut Mucosal Colonization Resistance to Empiric Probiotics Is Associated With Unique Host and Microbiome Features. Cell (2018) 174(6):1388–405:e21. 10.1016/j.cell.2018.08.041 30193112

[173] Stefan KL Kim MV Iwasaki A Kasper DL. Commensal Microbiota Modulation of Natural Resistance to Virus Infection. Cell (2020) 183(5):1312–24:e10. 10.1016/j.cell.2020.10.047 PMC7799371 33212011

[174] Pabst O Hornef M. Gut Microbiota: A Natural Adjuvant for Vaccination. Immunity (2014) 41(3):349–51. 10.1016/j.immuni.2014.09.002 25238091

[175] Kullberg MC Jankovic D Gorelick PL Caspar P Letterio JJ Cheever AW. Bacteria-Triggered CD4(+) T Regulatory Cells Suppress Helicobacter Hepaticus-Induced Colitis. J Exp Med (2002) 196(4):505–15. 10.1084/jem.20020556 PMC2196050 12186842

[176] Kuehl CJ Wood HD Marsh TL Schmidt TM Young VB. Colonization of the Cecal Mucosa by Helicobacter Hepaticus Impacts the Diversity of the Indigenous Microbiota. Infect Immun (2005) 73(10):6952–61. 10.1128/IAI.73.10.6852-6961.2005 PMC1230902 16177375

[177] Beura LK Hamilton SE Bi K Schenkel JM Odumade OA Casey KA. Normalizing the Environment Recapitulates Adult Human Immune Traits in Laboratory Mice. Nature (2016) 532(7600):512–6. 10.1038/nature17655 PMC4871315 27096360

[178] Siegrist CA Aspinall R. B-Cell Responses to Vaccination At the Extremes of Age. Nat Rev Immunol (2009) 9(3):185–94. 10.1038/nri2508 19240757

[179] Goronzy JJ Weyand CM. Understanding Immunosenescence to Improve Responses to Vaccines. Nat Immunol (2013) 14(5):428–36. 10.1038/ni.2588 PMC4183346 23598398

[180] Raymond SL Rincon JC Wynn JL Moldawer LL Larson SD. Impact of Early-Life Exposures to Infections, Antibiotics, and Vaccines on Perinatal and Long-term Health and Disease. Front Immunol (2017) 8:729. 10.3389/fimmu.2017.00729 28690615 PMC5481313

[181] Niewiesk S. Maternal Antibodies: Clinical Significance, Mechanism of Interference With Immune Responses, and Possible Vaccination Strategies. Front Immunol (2014) 5:446. 10.3389/fimmu.2014.00446 25278941 PMC4165321

[182] Church JA Parker EP Kirkpatrick BD Grassly NC Prendergast AJ. Interventions to Improve Oral Vaccine Performance: A Systematic Review and Meta-Analysis. Lancet Infect Dis (2019) 19(2):203–14. 10.1016/S1473-3099(18)30602-9 PMC6353819 30712836

[183] Timens W Boes A Rozeboom-Uiterwijk T Poppema S. Immaturity of the Human Splenic Marginal Zone in Infancy. Possible contribution to the deficient infant immune response. J Immunol (1989) 143(10):3200–6. 2478621

[184] Pihlgren M Tougne C Bozzotti P Fulurija A Duchosal MA Lambert PH. Unresponsiveness to Lymphoid-Mediated Signals At the Neonatal Follicular Dendritic Cell Precursor Level Contributes to Delayed Germinal Center Induction and Limitations of Neonatal Antibody Responses to T-dependent Antigens. J Immunol (2003) 170(6):2824–32. 10.4049/jimmunol.170.6.2824 12626532

[185] Kanswal S Katsenelson N Selvapandiyan A Bram RJ Akkoyunlu M. Deficient TACI Expression on B Lymphocytes of Newborn Mice Leads to Defective Ig Secretion in Response to BAFF or APRIL. J Immunol (2008) 181(2):976–90. 10.4049/jimmunol.181.2.976 18606649

[186] Garg M Luo W Kaplan AM Bondada S. Cellular Basis of Decreased Immune Responses to Pneumococcal Vaccines in Aged Mice. Infect Immun (1996) 64(11):4456–62. 10.1128/IAI.64.11.4456-4462.1996 PMC174398 8890192

[187] Asanuma H Hirokawa K Uchiyama M Suzuki Y Aizawa C Kurata T. Immune Responses and Protection in Different Strains of Aged Mice Immunized Intranasally With an Adjuvant-Combined Influenza Vaccine. Vaccine (2001) 19(28-29):3981–9. 10.1016/S0264-410X(01)00129-3 11427274

[188] Kang I Hong MS Nolasco H Park SH Dan JM Choi JY. Age-Associated Change in the Frequency of Memory CD4+ T Cells Impairs Long Term CD4+ T Cell Responses to Influenza Vaccine. J Immunol (2004) 173(1):673–81. 10.4049/jimmunol.173.1.673 15210831

[189] Wagar LE Gentleman B Pircher H McElhaney JE Watts TH. Influenza-Specific T Cells From Older People are Enriched in the Late Effector Subset and Their Presence Inversely Correlates With Vaccine Response. PloS One (2011) 6(8):e23698. 10.1371/journal.pone.0023698 21887299 PMC3161762

[190] Cummins NW Weaver EA May SM Croatt AJ Foreman O Kennedy RB. Heme Oxygenase-1 Regulates the Immune Response to Influenza Virus Infection and Vaccination in Aged Mice. FASEB J (2012) 26(7):2911–8. 10.1096/fj.11-190017 PMC3382093 22490782

[191] Haq K McElhaney JE. Immunosenescence: Influenza Vaccination and the Elderly. Curr Opin Immunol (2014) 29:38–42. 10.1016/j.coi.2014.03.008 24769424

[192] Nakaya HI Hagan T Duraisingham SS Lee EK Kwissa M Rouphael N. Systems Analysis of Immunity to Influenza Vaccination Across Multiple Years and in Diverse Populations Reveals Shared Molecular Signatures. Immunity (2015) 43(6):1186–98. 10.1016/j.immuni.2015.11.012 PMC4859820 26682988

[193] Arrieta MC Stiemsma LT Amenyogbe N Brown EM Finlay B. The Intestinal Microbiome in Early Life: Health and Disease. Front Immunol (2014) 5:427. 10.3389/fimmu.2014.00427 25250028 PMC4155789

[194] Nguyen QN Himes JE Martinez DR Permar SR. The Impact of the Gut Microbiota on Humoral Immunity to Pathogens and Vaccination in Early Infancy. PloS Pathog (2016) 12(12):e1005997. 10.1371/journal.ppat.1005997 28006021 PMC5179050

[195] Gonzalez-Perez G Hicks AL Tekieli TM Radens CM Williams BL Lamouse-Smith ES. Maternal Antibiotic Treatment Impacts Development of the Neonatal Intestinal Microbiome and Antiviral Immunity. J Immunol (2016) 196(9):3768–79. 10.4049/jimmunol.1502322 27036912

[196] Al Nabhani Z Eberl G. Imprinting of the Immune System by the Microbiota Early in Life. Mucosal Immunol (2020) 13(2):183–9. 10.1038/s41385-020-0257-y 31988466

[197] Arrieta MC Stiemsma LT Dimitriu PA Thorson L Russell S Yurist-Doutsch S. Early Infancy Microbial and Metabolic Alterations Affect Risk of Childhood Asthma. Sci Transl Med. (2015) 7(307):307ra152. 10.1126/scitranslmed.aab2271 26424567

[198] Rampelli S Candela M Turroni S Biagi E Collino S Franceschi C. Functional Metagenomic Profiling of Intestinal Microbiome in Extreme Ageing. Aging (Albany NY) (2013) 5(12):902–12. 10.18632/aging.100623 PMC3883706 24334635

[199] Salazar N Valdes-Varela L Gonzalez S Gueimonde M de Los Reyes-Gavilan CG. Nutrition and the Gut Microbiome in the Elderly. Gut Microbes (2017) 8(2):82–97. 10.1080/19490976.2016.1256525 27808595 PMC5390822

[200] Amsterdam D Ostrov BE. The Impact of the Microbiome on Immunosenescence. Immunol Invest (2018) 47(8):801–11. 10.1080/08820139.2018.1537570 31282802

[201] Poland GA Ovsyannikova IG Kennedy RB Lambert ND Kirkland JL. A Systems Biology Approach to the Effect of Aging, Immunosenescence and Vaccine Response. Curr Opin Immunol (2014) 29:62–8. 10.1016/j.coi.2014.04.005 PMC4119552 24820347

[202] Murray MA Chotirmall SH. The Impact of Immunosenescence on Pulmonary Disease. Mediators Inflammation (2015) 2015:692546. 10.1155/2015/692546 PMC4495178 26199462

[203] Maidens C Childs C Przemska A Dayel IB Yaqoob P. Modulation of Vaccine Response by Concomitant Probiotic Administration. Br J Clin Pharmacol (2013) 75(3):663–70. 10.1111/j.1365-2125.2012.04404.x PMC3575933 22845346

[204] Lei WT Shih PC Liu SJ Lin CY Yeh TL. Effect of Probiotics and Prebiotics on Immune Response to Influenza Vaccination in Adults: A Systematic Review and Meta-Analysis of Randomized Controlled Trials. Nutrients (2017) 9(11):1175. 10.3390/nu9111175 PMC5707647 29077061

[205] Zimmermann P Curtis N. The Influence of Probiotics on Vaccine Responses - A Systematic Review. Vaccine (2018) 36(2):207–13. 10.1016/j.vaccine.2017.08.069 28923425

[206] Yeh TL Shih PC Liu SJ Lin CH Liu JM Lei WT. The Influence of Prebiotic or Probiotic Supplementation on Antibody Titers After Influenza Vaccination: A Systematic Review and Meta-Analysis of Randomized Controlled Trials. Drug Des Devel Ther (2018) 12:217–30. 10.2147/DDDT.S155110 PMC5790137 29416317

[207] Praharaj I John SM Bandyopadhyay R Kang G. Probiotics, Antibiotics and the Immune Responses to Vaccines. Philos Trans R Soc Lond B Biol Sci (2015) 370(1671). 10.1098/rstb.2014.0144 PMC4527389 25964456

[208] Olivares M Diaz-Ropero MP Sierra S Lara-Villoslada F Fonolla J Navas M. Oral Intake of Lactobacillus Fermentum CECT5716 Enhances the Effects of Influenza Vaccination. Nutrition (2007) 23(3):254–60. 10.1016/j.nut.2007.01.004 17352961

[209] Bianchini S Orabona C Camilloni B Berioli MG Argentiero A Matino D. Effects of Probiotic Administration on Immune Responses of Children and Adolescents With Type 1 Diabetes to a Quadrivalent Inactivated Influenza Vaccine. Hum Vaccin Immunother (2020) 16(1):86–94. 10.1080/21645515.2019.1633877 31210557 PMC7012143

[210] Taylor AL Hale J Wiltschut J Lehmann H Dunstan JA Prescott SL. Effects of Probiotic Supplementation for the First 6 Months of Life on Allergen- and Vaccine-Specific Immune Responses. Clin Exp Allergy (2006) 36(10):1227–35. 10.1111/j.1365-2222.2006.02553.x 17014429

[211] Redondo N Nova E Gheorghe A Diaz LE Hernandez A Marcos A. Evaluation of Lactobacillus Coryniformis CECT5711 Strain as a Coadjuvant in a Vaccination Process: A Randomised Clinical Trial in Healthy Adults. Nutr Metab (Lond) (2017) 14:2. 10.1186/s12986-017-0193-3 28070204 PMC5217323

[212] Bunout D Barrera G Hirsch S Gattas V de la Maza MP Haschke F. Effects of a Nutritional Supplement on the Immune Response and Cytokine Production in Free-Living Chilean Elderly. JPEN J Parenter Enteral Nutr (2004) 28(5):348–54. 10.1177/0148607104028005348 15449576

[213] Rizzardini G Eskesen D Calder PC Capetti A Jespersen L Clerici M. Evaluation of the Immune Benefits of Two Probiotic Strains Bifidobacterium Animalis Ssp. Lactis, BB-12(R) and Lactobacillus Paracasei Ssp. Paracasei, L. Casei 431(R) in an Influenza Vaccination Model: A Randomised, Double-Blind, Placebo-Controlled Study. Br J Nutr (2012) 107(6):876–84. 10.1017/S000711451100420X 21899798

[214] Mercenier A Muller-Alouf H Grangette C. Lactic Acid Bacteria as Live Vaccines. Curr Issues Mol Biol (2000) 2(1):17–25. 11464916

[215] Szatraj K Szczepankowska AK Chmielewska-Jeznach M. Lactic Acid Bacteria - Promising Vaccine Vectors: Possibilities, Limitations, Doubts. J Appl Microbiol (2017) 123(2):325–39. 10.1111/jam.13446 PMC7166332 28295939

[216] Wells JM. Immunomodulatory Mechanisms of Lactobacilli. Microb Cell Fact (2011) 10 Suppl 1:S17. 10.1186/1475-2859-10-S1-S17 21995674 PMC3231924

[217] Bunout D Hirsch S Pia de la Maza M Munoz C Haschke F Steenhout P. Effects of Prebiotics on the Immune Response to Vaccination in the Elderly. JPEN J Parenter Enteral Nutr (2002) 26(6):372–6. 10.1177/0148607102026006372 12405649

[218] Duggan C Penny ME Hibberd P Gil A Huapaya A Cooper A. Oligofructose-Supplemented Infant Cereal: 2 Randomized, Blinded, Community-Based Trials in Peruvian Infants. Am J Clin Nutr (2003) 77(4):937–42. 10.1093/ajcn/77.4.937 12663295

[219] Salvini F Riva E Salvatici E Boehm G Jelinek J Banderali G. A Specific Prebiotic Mixture Added to Starting Infant Formula has Long-Lasting Bifidogenic Effects. J Nutr (2011) 141(7):1335–9. 10.3945/jn.110.136747 PMC3113290 21613452

[220] Stam J van Stuijvenberg M Garssen J Knipping K Sauer PJ. A Mixture of Three Prebiotics Does Not Affect Vaccine Specific Antibody Responses in Healthy Term Infants in the First Year of Life. Vaccine (2011) 29(44):7766–72. 10.1016/j.vaccine.2011.07.110 21821078

[221] van den Berg JP Westerbeek EA van der Klis FR Berbers GA Lafeber HN van Elburg RM. Neutral and Acidic Oligosaccharides Supplementation Does Not Increase the Vaccine Antibody Response in Preterm Infants in a Randomized Clinical Trial. PloS One (2013) 8(8):e70904. 10.1371/journal.pone.0070904 23951035 PMC3738516

[222] Akatsu H Nagafuchi S Kurihara R Okuda K Kanesaka T Ogawa N. Enhanced Vaccination Effect Against Influenza by Prebiotics in Elderly Patients Receiving Enteral Nutrition. Geriatr Gerontol Int (2016) 16(2):205–13. 10.1111/ggi.12454 25613751

[223] Medina M Izquierdo E Ennahar S Sanz Y. Differential Immunomodulatory Properties of Bifidobacterium Logum Strains: Relevance to Probiotic Selection and Clinical Applications. Clin Exp Immunol (2007) 150(3):531–8. 10.1111/j.1365-2249.2007.03522.x PMC2219384 17956582

[224] Rooks MG Garrett WS. Gut Microbiota, Metabolites and Host Immunity. Nat Rev Immunol (2016) 16(6):341–52. 10.1038/nri.2016.42 PMC5541232 27231050

[225] Shibata N Kunisawa J Kiyono H. Dietary and Microbial Metabolites in the Regulation of Host Immunity. Front Microbiol (2017) 8:2171. 10.3389/fmicb.2017.02171 29163449 PMC5681998

[226] Ratajczak W Ryl A Mizerski A Walczakiewicz K Sipak O Laszczynska M. Immunomodulatory Potential of Gut Microbiome-Derived Short-Chain Fatty Acids (Scfas). Acta Biochim Pol (2019) 66(1):1–12. 10.18388/abp.2018_2648 30831575

[227] Nomura M Nagatomo R Inoue K Doi K Shimizu J Baba K. 1249p Association of SCFA in Gut Microbiome and Clinical Response in Solid Cancer Patients Treated With andi-PD-1 Antibody. Ann Oncol (2019) 30(Supplement_5):mdz253.074. 10.1093/annonc/mdz253.074

[228] Sim JR Kang SS Lee D Yun CH Han SH. Killed Whole-Cell Oral Cholera Vaccine Induces Ccl20 Secretion by Human Intestinal Epithelial Cells in the Presence of the Short-Chain Fatty Acid, Butyrate. Front Immunol (2018) 9:55. 10.3389/fimmu.2018.00055 29434590 PMC5796904

[229] Guo CJ Allen BM Hiam KJ Dodd D Van Treuren W Higginbottom S. Depletion of Microbiome-Derived Molecules in the Host Using Clostridium Genetics. Science (2019) 366(6471):eaav1282. 10.1126/science.aav1282 31831639 PMC7141153

[230] Arpaia N Campbell C Fan X Dikiy S van der Veeken J deRoos P. Metabolites Produced by Commensal Bacteria Promote Peripheral Regulatory T-cell Generation. Nature (2013) 504(7480):451–5. 10.1038/nature12726 PMC3869884 24226773

[231] Thorburn AN McKenzie CI Shen S Stanley D Macia L Mason LJ. Evidence That Asthma is a Developmental Origin Disease Influenced by Maternal Diet and Bacterial Metabolites. Nat Commun (2015) 6:7320. 10.1038/ncomms8320 26102221

[232] Maslowski KM Vieira AT Ng A Kranich J Sierro F Yu D. Regulation of Inflammatory Responses by Gut Microbiota and Chemoattractant Receptor GPR43. Nature (2009) 461(7268):1282–6. 10.1038/nature08530 PMC3256734 19865172

[233] Singh N Gurav A Sivaprakasam S Brady E Padia R Shi H. Activation of Gpr109a, Receptor for Niacin and the Commensal Metabolite Butyrate, Suppresses Colonic Inflammation and Carcinogenesis. Immunity (2014) 40(1):128–39. 10.1016/j.immuni.2013.12.007 PMC4305274 24412617

[234] Macia L Tan J Vieira AT Leach K Stanley D Luong S. Metabolite-Sensing Receptors GPR43 and GPR109A Facilitate Dietary Fibre-Induced Gut Homeostasis Through Regulation of the Inflammasome. Nat Commun (2015) 6:6734. 10.1038/ncomms7734 25828455

[235] Erny D Hrabe de Angelis AL Jaitin D Wieghofer P Staszewski O David E. Host Microbiota Constantly Control Maturation and Function of Microglia in the CNS. Nat Neurosci (2015) 18(7):965–77. 10.1038/nn.4030 PMC5528863 26030851

[236] Parker LC Prince LR Sabroe I. Translational Mini-Review Series on Toll-like Receptors: Networks Regulated by Toll-like Receptors Mediate Innate and Adaptive Immunity. Clin Exp Immunol (2007) 147(2):199–207. 10.1111/j.1365-2249.2006.03203.x 17223959 PMC1810480

[237] Hua Z Hou B. TLR Signaling in B-cell Development and Activation. Cell Mol Immunol (2013) 10(2):103–6. 10.1038/cmi.2012.61 PMC4003046 23241902

[238] Jin B Sun T Yu XH Yang YX Yeo AE. The Effects of TLR Activation on T-cell Development and Differentiation. Clin Dev Immunol (2012) 2012:836485. 10.1155/2012/836485 22737174 PMC3376488

[239] Heer AK Shamshiev A Donda A Uematsu S Akira S Kopf M. TLR Signaling Fine-Tunes Anti-Influenza B Cell Responses Without Regulating Effector T Cell Responses. J Immunol (2007) 178(4):2182–91. 10.4049/jimmunol.178.4.2182 17277123

[240] Meyer-Bahlburg A Khim S Rawlings DJ. B Cell Intrinsic TLR Signals Amplify But are Not Required for Humoral Immunity. J Exp Med (2007) 204(13):3095–101. 10.1084/jem.20071250 PMC2150979 18039950

[241] Salerno F Freen-van Heeren JJ Guislain A Nicolet BP Wolkers MC. Costimulation Through TLR2 Drives Polyfunctional Cd8(+) T Cell Responses. J Immunol (2019) 202(3):714–23. 10.4049/jimmunol.1801026 30578304

[242] McCarron M Reen DJ. Activated Human Neonatal CD8+ T Cells are Subject to Immunomodulation by Direct TLR2 or TLR5 Stimulation. J Immunol (2009) 182(1):55–62. 10.4049/jimmunol.182.1.55 19109135

[243] Jun JC Jones MB Oswald DM Sim ES Jonnalagadda AR Kreisman LSC. T Cell-Intrinsic TLR2 Stimulation Promotes IL-10 Expression and Suppressive Activity by CD45RbHi T Cells. PloS One (2017) 12(7):e0180688. 10.1371/journal.pone.0180688 28742882 PMC5526543

[244] Li Q Yan Y Liu J Huang X Zhang X Kirschning C. Toll-Like Receptor 7 Activation Enhances Cd8+ T Cell Effector Functions by Promoting Cellular Glycolysis. Front Immunol (2019) 10:2191. 10.3389/fimmu.2019.02191 31572396 PMC6751247

[245] Komai-Koma M Jones L Ogg GS Xu D Liew FY. TLR2 is Expressed on Activated T Cells as a Costimulatory Receptor. Proc Natl Acad Sci USA (2004) 101(9):3029–34. 10.1073/pnas.0400171101 PMC365739 14981245

[246] Gringhuis SI Kaptein TM Wevers BA van der Vlist M Klaver EJ van Die I. Fucose-Based PAMPs Prime Dendritic Cells for Follicular T Helper Cell Polarization Via DC-SIGN-dependent Il-27 Production. Nat Commun (2014) 5:5074. 10.1038/ncomms6074 25278262

[247] Gringhuis SI Kaptein TM Wevers BA Mesman AW Geijtenbeek TB. Fucose-Specific DC-SIGN Signalling Directs T Helper Cell Type-2 Responses Via IKKepsilon- and CYLD-dependent Bcl3 Activation. Nat Commun (2014) 5:3898. 10.1038/ncomms4898 24867235

[248] Levy M Thaiss CA Zeevi D Dohnalova L Zilberman-Schapira G Mahdi JA. Microbiota-Modulated Metabolites Shape the Intestinal Microenvironment by Regulating Nlrp6 Inflammasome Signaling. Cell (2015) 163(6):1428–43. 10.1016/j.cell.2015.10.048 PMC5665753 26638072

[249] Ishiguro K Ando T Maeda O Watanabe O Goto H. Suppressive Action of Acetate on Interleukin-8 Production Via Tubulin-Alpha Acetylation. Immunol Cell Biol (2014) 92(7):624–30. 10.1038/icb.2014.31 24777307

